# Chemical proteomics reveals post-translational remodeling of endosomal pathways during macrophage activation

**DOI:** 10.64898/2026.09.25.754469

**Published:** 2026-10-01

**Authors:** Tiffany L. Zhang, Henry Sanford, Railia Gabbasova, Anne E. van Vlimmeren, Yannis Kalaidzidis, Hiroyuki Kojima, Charlotte R. Wayne, Jeetayu Biswas, Priyam Banerjee, Beatrice Zhang, Omar Abdel-Wahab, Marino Zerial, Ekaterina V. Vinogradova

**Affiliations:** 1 Department of Chemical Immunology and Proteomics, The Rockefeller University, New York, New York, 10065, USA.; 2 Tri-Institutional PhD Program in Chemical Biology, New York, New York, 10021, USA.; 3 Max Planck Institute of Molecular Cell Biology and Genetics, Dresden, Germany.; 4 Molecular Pharmacology Program, Sloan Kettering Institute, Memorial Sloan Kettering Cancer Center, New York, New York, 10065, USA.; 5 Leukemia Service, Department of Medicine, Memorial Sloan Kettering Cancer Center, New York, New York, 10065, USA.; 6 Bio-Imaging Resource Center, The Rockefeller University, New York, New York, 10065, USA.; 7 Human Technopole, 20157 Milan, Italy.

## Abstract

Macrophages are central regulators of innate immunity, yet the post-translational mechanisms that enable rapid inflammatory responses remain incompletely understood. Here, we integrated transcriptomics, unenriched proteomics, phosphoproteomics, metabolomics, and cysteine reactivity profiling to comprehensively characterize primary human macrophages following Toll-like receptor and Stimulator of Interferon Genes (STING) activation. This approach revealed substantial discordance between transcript- and protein-level changes, as well as extensive post-translational remodeling. Differentially reactive cysteines were enriched in proteins involved in small-GTPase signaling, vesicular trafficking, and the endo-lysosomal system. Reactivity changes in two subunits of the endosomal mRNA-transport FERRY complex nominated it as a candidate target of inflammation-associated post-translational regulation. Biochemical and imaging analyses established FERRY complex assembly and early endosomal localization in primary human macrophages and identified DISC1 as a previously unrecognized FERRY-associated protein. This work provides a comprehensive resource of biochemical and post-translational remodeling during macrophage activation and implicates endosome-associated mRNA transport in innate immune regulation.

## INTRODUCTION

Macrophages coordinate innate immune responses by integrating environmental cues to regulate antigen presentation, cytokine production, and phagocytosis^1^. Toll-like receptors (TLRs)^2^ and the stimulator of interferon genes (STING) pathway^3^ are complementary innate immune sensors that detect diverse pathogen- and damage-associated molecular patterns. Activation of these pathways represents a central mechanism driving pro-inflammatory macrophage signaling. Dysregulation of TLR and STING signaling has been implicated in numerous human diseases^4, 5^. Autoimmune disorders, including systemic lupus erythematosus and rheumatoid arthritis^6^, are characterized by elevated levels of endogenous TLR ligands, whereas defects in downstream TLR signaling components have been linked to increased susceptibility to infection, attenuated innate immune responses, and impaired T cell activation^7^.

Despite the central role of these pathways in host defense and disease, their molecular characterization has relied heavily on immortalized cell lines and mouse models that fail to fully recapitulate the biology of primary human macrophages^8, 9, 10^. Furthermore, most studies have focused on transcriptomic profiling, even though mRNA levels often correlate poorly with protein abundance^11, 12, 13, 14^. Protein function is further regulated through post-translational mechanisms that play a role in their activity, localization, and molecular interactions. As a result, abundance-based proteomic analyses alone provide an incomplete picture of the biochemical changes that control macrophage activation.

To address this gap, we generated a comprehensive multi-omic atlas of TLR- and STING-activated primary human macrophages by integrating transcriptomics, abundance-based and functional proteomics, and metabolomics. Central to this analysis was tandem mass tag-enabled activity-based protein-profiling (TMT-ABPP)^15, 16^, which uses cysteine-reactive probes to detect structural and functional changes in protein states independently of protein abundance. Previous studies have demonstrated that this approach captures proteome remodeling associated with phosphorylation^17^, redox signaling during T cell activation^15^, metabolic dysfunction associated with T cell exhaustion^18^, and lysosomal trafficking in macrophages infected with *Mycobacterium tuberculosis*^19^. In dysfunctional T cells, this approach also identified broader protein networks sensitive to ATP availability^18^, highlighting the capacity of reactive cysteines to serve as sensitive reporters of cellular biochemical state beyond changes in protein abundance.

Here, we applied this integrated strategy to characterize diverse pro-inflammatory states induced by TLR and STING agonists in primary human macrophages. The resulting dataset revealed extensive proteome remodeling, including coordinated changes in small GTPase networks, vesicle trafficking machinery, and phosphoinositide signaling across the endo-lysosomal system. Among the state-dependent targets, we identified two subunits of FERRY, a recently described Rab5 effector complex that mediates endosomal mRNA transport and localized translation in neurons^20, 21^. We further demonstrated FERRY complex assembly and TLR4-dependent remodeling in macrophages, pointing to a previously unrecognized role of endosome-associated mRNA transport and localized translation in macrophage biology.

## RESULTS

## A scalable primary human macrophage platform enables multi-omic profiling of TLR and STING activation

We developed a robust and scalable *in vitro* differentiation platform to characterize molecular programs associated with TLR and STING signaling in primary human macrophages. Briefly, CD14^+^ monocytes isolated from peripheral blood mononuclear cells (PBMCs) of healthy donors were differentiated into resting (M0) macrophages over six days in the presence of macrophage colony stimulating factor (mCSF), followed by stimulation with TLR ligands or a STING agonist for 24 hours before downstream analyses (Fig. 1a). This platform enables generation of primary human macrophages, supporting integrated multi-omic profiling of macrophage activation, including transcriptomics, abundance-based proteomics, cysteine reactivity profiling, phosphoproteomics, and metabolomics.

We validated this platform by assessing macrophage activation using flow cytometry analysis of cell-surface markers and quantification of inflammatory cytokine secretion by ELISA (Fig. 1b,c and Extended Data Fig. 1a). Stimulation of multiple TLR pathways resulted in reduced surface levels of CD14, consistent with the reported endocytosis of this receptor following activation^22^. CD11b remained relatively unchanged, while the costimulatory receptor CD80 was upregulated in most stimulation conditions (Fig. 1b and Extended Data Fig. 1a). In parallel, we observed elevated secretion of pro-inflammatory cytokines IL-6 and TNF, canonical downstream effectors of TLR signaling in macrophages^23^ (Fig. 1c). These results confirmed robust activation of primary human macrophages across the stimulation conditions, with the notable exception of TLR9 stimulation, which did not show altered surface marker expression or cytokine production relative to M0 cells.

### TLR and STING activation drive extensive inflammatory and metabolic remodeling in primary human macrophages

To comprehensively characterize molecular programs associated with macrophage activation, we profiled transcriptional and proteomic changes using RNA-sequencing and abundance-based proteomics with tandem mass tag-enabled sample multiplexing (TMT-exp; Fig. 1d and Extended Data Fig. 1b,c; Data S1–1, S2–1, S2–2). Transcriptional responses were generally more pronounced than corresponding changes in protein abundance, underscoring the contribution of post-transcriptional mechanisms to macrophage activation and highlighting the importance of protein-level analyses for capturing functionally relevant transitions in macrophage states. Consistent with our immune profiling experiments, TLR9 agonism produced minimal transcriptional and protein level changes, likely reflecting low TLR9 expression in mCSF-differentiated macrophages. Consistent with this, TLR9 itself was not quantified in our RNA-sequencing and abundance-based proteomics experiments, and this condition was therefore excluded from subsequent analyses.

Principal component analysis of both transcriptomic and proteomic profiles revealed clear separation of TLR- and STING-stimulated macrophages from resting M0 cells (Extended Data Fig. 1b,c). Within the TLR conditions, TLR3- and TLR4-stimulated macrophages clustered separately from other TLR-stimulated conditions, consistent with their ability to engage TRIF-dependent signaling and induce type I interferon responses, in contrast to the predominantly MyD88-dependent pathways used by other TLRs^23^. STING activation, which also promotes type I interferon production through a mechanistically distinct pathway, generated a discrete transcriptional and proteomic signature, clustering separately from both TLR-stimulated and resting macrophages, highlighting the diversity of pro-inflammatory states captured within this dataset.

Gene set enrichment analysis (GSEA) of proteins contributing to the principal component 1 (PC1) loadings revealed upregulation of pro-inflammatory and antiviral programs and downregulation of aerobic metabolic pathways (Extended Data Fig. 1d; Data S2–3). Principal component 2 (PC2) distinguished STING activation from other TLR-stimulated conditions, highlighting the unique molecular features of the STING response. Consistent with these findings, GSEA demonstrated broad activation of TNF signaling across all inflammatory states, while interferon-responsive programs were preferentially enriched following STING, TLR3, and TLR4 stimulation (Extended Data Fig. 1e,f).

Upon activation, macrophages undergo metabolic reprogramming characterized by a shift from oxidative phosphorylation (OXPHOS) to aerobic glycolysis^25^. This transition supports rapid ATP generation and is accompanied by remodeling of the tricarboxylic acid (TCA) cycle, leading to accumulation of immunoregulatory metabolites, including citrate^26^, succinate^27, 28^, and itaconate^29, 30^ (Fig. 1e). In parallel, increased glycolytic flux and diversion of glucose intermediates into the pentose phosphate pathway promote NADPH production and reactive oxygen species (ROS) generation^25^. Consistent with this, we observed increased expression of glycolytic enzymes across all stimulation conditions, together with reduced abundance of multiple electron transport chain (ETC) components and ATP synthase subunits (Fig. 1f,g, Extended Data Fig. 1g-i).

Notably, macrophage activation was associated with broad suppression of mitochondrial translation, in contrast to the more heterogeneous regulation of other mitochondrial proteins (Fig. 1g and Extended Data Fig. 1i). Downregulated proteins included mitochondrial ribosome-associated GTPases (ERAL1), components of mitochondrial tRNA processing complexes (PTCD1, QRSL1, GATC), and numerous mitochondrial ribosomal subunits. Since mitochondria encode and synthesize key subunits of the ETC, coordinated suppression of ETC components and mitochondrial translation may enable macrophages to rapidly downregulate OXPHOS during inflammation. These findings underscore the extensive metabolic remodeling that is required during TLR- and STING-mediated macrophage activation and the establishment of distinct inflammatory cell states.

### Post-transcriptional regulation contributes to proteome remodeling during macrophage activation

We next compared transcript- and protein-level changes to identify proteins subject to post-transcriptional regulation and define factors contributing to this divergence (Fig. 1h, Extended Data Fig. 2a; Data S2–2). In TLR4-stimulated macrophages, we observed only moderate correlation between mRNA and protein abundance (R = 0.42; Fig. 1h). Similar correlations were found for other stimulation conditions in macrophages (Extended Data Fig. 2a), in line with our observations in activated and exhausted T cells^15, 18^. This analysis identified proteins whose abundance closely tracked transcript levels (grey), as well as proteins regulated predominantly at either the transcript (purple) or protein (yellow) level, in addition to a subset of proteins displaying anti-correlated expression patterns (teal) (Fig. 1h).

Gene ontology analysis of proteins in the teal (anti-correlated) and yellow (regulated at the protein-level) categories revealed enrichment of metabolic, signaling, and mitochondrial processes, including glycolysis (PKM, PFKL, ENO1) and mitochondrial translation (mitochondrial ribosomes and aminoacyl-tRNA synthetases) (Fig. 1h, Extended Data Fig. 2b; Data S2–4). The post-transcriptional regulation of these pathways may enable rapid adaptation to inflammatory and metabolic stress during macrophage activation. We validated representative targets by qPCR and Western blot analysis, including SCO2, CAMK1, and S100A4 (teal), as well as STAT4 (grey) (Extended Data Fig. 2c-e).

Our findings indicate that transcriptional regulation accounts for only a subset of the proteomic remodeling that accompanies macrophage activation, suggesting additional layers of post-translational regulation that shape inflammatory protein landscapes. To test whether differences in protein turnover also contribute to the observed protein-transcript discordance, we integrated published protein half-life measurements from primary human monocytes^24^. Proteins selectively downregulated following TLR4 stimulation at the protein level (teal) were enriched for short-lived species, with relatively few exhibiting half-lives greater than 48 h (Fig. 1i, top). Conversely, proteins selectively upregulated at the protein level tended to be longer lived, with fewer than 20% displaying half-lives below 48 h (Fig. 1i, bottom). This trend was conserved across all stimulation conditions (Extended Data Fig. 2f), suggesting that protein turnover may serve as an additional layer of regulation of protein abundance and ultimately, macrophage activation states.

### Cysteine reactivity profiling reveals extensive biochemical and post-translational remodeling during macrophage activation

To capture additional layers of post-translational regulation during macrophage activation, we applied TMT-ABPP^15, 16^, a multiplexed chemical proteomic platform that uses cysteine reactivity toward electrophilic probes as a readout of biochemical changes in distinct cellular states (Fig. 2a; Data S2–5). The broadly reactive iodoacetamide-desthiobiotin (IA-DTB) probe covalently labels a subset of cysteine residues, which can then be selectively enriched for downstream proteomic analysis. Probe reactivity is sensitive to post-translational modifications (PTMs)^17^, changes in redox state^15^, protein-metabolite and protein-protein interactions (PPIs)^18^, while enrichment of cysteine-reactive peptides can also be indirectly affected through modifications of adjacent amino acids within the same tryptic peptide^17^. Thus, TMT-ABPP provides an unbiased and orthogonal strategy for identifying different forms of biochemical and post-translational regulation that shape macrophage activation states.

We compared cysteine reactivity across resting (M0), TLR-, and STING-stimulated macrophages using a 16-plex TMT-ABPP workflow (Fig. 2a,b; Data S2–5). To identify robust reactivity changes independent of protein abundance changes, we applied a stringent set of filters, restricting analysis to proteins with at least two quantified cysteine-containing peptides and reactivity changes exceeding twofold relative to the corresponding change in protein abundance (See Methods). Across all stimulation conditions, we identified 495 reactivity changes, with TLR3 and TLR4 stimulation producing the largest number of changes (Fig. 2b). Consistent with distinct cell-type-specific signaling programs, overlap in cysteine reactivity changes between macrophages and activated T cells^15^ was limited, likely reflecting differences in both protein expression and the underlying biology of these immune cell populations (Extended Data Fig. 3a,b).

### Chemoproteomic profiling reveals dynamic remodeling of GTPase signaling in activated macrophages

TLR4 stimulation produced the largest number of cysteine reactivity changes (Fig. 2b) and was therefore chosen for subsequent analyses. GO biological process (GO:BP) analysis of proteins with reactivity changes following TLR4 stimulation identified GTPase-mediated signal transduction as the most enriched term (Fig. 2c; Data S2–4). GTPases are central regulators of macrophage function, controlling inflammatory signaling, cytoskeletal dynamics, phagocytosis, and intracellular trafficking.^32, 33, 34, 35^ Closer inspection revealed >30 changes in GTPases, as well as in guanine nucleotide exchange factors (GEFs) and GTPase activating proteins (GAPs), which dynamically control GTPase activity (Fig. 2d).

GAPs and GEFs are extensively regulated by PTMs, PPIs, and second messenger binding. These regulatory events are frequently coupled to conformational changes^36, 37^ that can alter cysteine accessibility or environment, consistent with the reactivity changes observed in our study. Several of these mapped to domains with established functional roles, including PIP_3_-binding domains within the ArfGAP ARAP1^38^ and the RacGEF TIAM1^39^, with PIP_3_ binding known to promote their catalytic activity (Fig. 2d). Other changes, including those in RabGEFs RAB3IL1 and DENND4C and the ArfGAP GIT1, occur in disordered regions adjacent to known phosphorylation sites, suggesting potential regulation through phosphorylation.

We also identified reactivity changes within the nucleotide-binding pockets of several GTPases, including CDC42 (Fig. 2e,f) and Rab12 (Extended Data Fig. 3c,d), suggesting that these changes may reflect GTP or GDP occupancy. CDC42 is a Rho family GTPase that regulates endocytosis, cytoskeletal remodeling and other cellular processes^40^. To test whether CDC42 cysteine reactivity is sensitive to guanine nucleotide binding, we performed *in vitro* GTP-addback experiments in lysates from M0 and TLR4-stimulated macrophages using a high molecular weight cysteine-reactive maleimide probe and monitored probe-induced band shifts by Western blot analysis. Probe labeling produced an upward shift in CDC42 migration, which was selectively blocked by GTP, but not ATP (Fig. 2g,h).

Notably, the GTP-sensitive band shift was more pronounced in M0 lysates compared to TLR4-stimulated lysates (Fig. 2g,h), consistent with a stimulation-dependent difference in CDC42 occupancy or regulatory state. We also observed reactivity changes in multiple CDC42 regulators, including ARHGEF7 and DOCK4/7, as well as in the CDC42 effector TNK2 (Extended Data Fig. 3e), suggesting remodeling of this pathway following TLR4 stimulation. Complementary metabolomic profiling of polar metabolites (Fig. 2i, Extended Data Fig. 3f, Data S3–1) revealed no change in intracellular GTP levels following TLR4 stimulation. This data suggests that the altered cysteine reactivity is unlikely to result from greater GTP availability and may instead reflect regulatory changes in nucleotide loading, conformation, or protein interactions of CDC42.

### Reactivity profiling reveals remodeling of vesicular trafficking and autophagy

GO:BP analysis revealed enrichment of proteins with reactivity changes in vesicular trafficking pathways in TLR4-stimulated macrophages (Fig. 3a; Data S2–4), including Golgi organization and macroautophagy. GO cellular component analysis (GO:CC) localized many of these changes to defined membrane-associated compartments, including early endosomes and autophagosomes (Fig. 3b). The proteins with reactivity changes span diverse roles in vesicle trafficking, including anterograde and retrograde transport, Golgi organization, vesicle tethering and fusion, and cytoskeletal regulation (Fig. 3c,d).

Several reactivity changes mapped to functionally important structural domains, including AP3D1_C574, located at the interface of AP3B3 within the AP3 adaptor complex^41^, and C347/C348 in the BAR domain of SNX6^42^, a component of the retromer complex, which heteromerizes with SNX1 or SNX2 (Fig. 3d,e). We also identified reactivity changes in multiple core regulators of autophagy, including ATG9A, ATG7, and UVRAG, consistent with the established role of TLR signaling in autophagy^18^ (Extended Data Fig. 4a,b).

Membrane trafficking is coordinated by phosphatidylinositol lipids, which define organelle identity and direct protein recruitment through the concerted actions of lipid kinases and phosphatases^45^ (Extended Data Fig. 4c). Consistent with extensive remodeling of intracellular membrane compartments following TLR activation, we observed multiple reactivity changes in phosphatidylinositol regulators, including decreased reactivity of C312 in PIP5K1A following TLR3 and TLR4 stimulation (Extended Data Fig. 4d). This lipid kinase generates PI(4,5)P_2_ at the plasma membrane and nucleus and requires membrane recruitment and dimerization for functional activation^46, 47^. The differentially reactive cysteine C312 lies at the protein homodimer interface^46^ (Extended Data Fig. 4d,e), which is consistent with reported activation of this kinase under TLR4 stimulation conditions to modulate cytoskeletal activity at phagocytic cups^48^.

### Phosphoproteomic analysis provides complementary information on post-translational regulation in activated macrophages

Considering vesicular trafficking is a post-translationally regulated process^49, 50, 51^, we complemented our reactivity profiling with phosphoproteomics analysis of M0 and TLR4-stimulated macrophages (Fig. 3f; Data S2–6). Using a dual phosphopeptide enrichment workflow with TiO_2_ and Fe-NTA, we quantified more than 9,000 phosphosites across 2,498 proteins (Fig. 3f and Extended Data Fig. 5a-c). GO:BP analysis of phosphosites with altered abundance following TLR4 stimulation revealed significant enrichment of pathways involved in endosomal transport (Fig. 3g, Extended Data Fig. 5d-f, Data S2–4), GTPase-mediated signal transduction (Extended Data Fig. 5g,h), and vesicle-mediated transport (Extended Data Fig. 5i-k), among others. Notably, the proteins contributing to these pathways in the phosphoproteomic dataset were largely distinct from those identified by expression-based proteomics (TMT-exp) or cysteine reactivity profiling (TMT-ABPP) (Fig. 3h, Extended Data Fig. 5e-k), highlighting how these orthogonal approaches reveal complementary information on post-translational regulation of these pathways. Finally, immunofluorescence (IF) analysis of the subcellular localization and distribution of the early endosomal marker Rab5 confirmed increased abundance of early endosomes following TLR4 stimulation (Fig. 3i-k).

### Chemoproteomic profiling identifies FERRY complex as a post-translationally regulated target in activated macrophages

To prioritize cysteine reactivity changes most likely to reflect biologically relevant regulatory events, we examined the structural and evolutionary properties of differentially reactive cysteines (Data S2–5). Relative to all quantified cysteines, differentially reactive cysteines were enriched in solvent-accessible and intrinsically disordered regions predicted by AlphaFold2^52^ (Fig. 4a,b, Extended Data Fig. 6a,b) with a smaller fraction of these sites predicted to be pathogenic by AlphaMissense^53^. Most of the cysteines were also highly conserved across species in both reactive and nonreactive groups^54^. Notably, nearly half of the proteins with differentially reactive cysteines were annotated as components of known protein complexes (CORUM^55, 56^ and Complex Portal^57, 58^; Extended Data Fig. 6a), suggesting that many of the observed reactivity changes may reflect state-dependent structural rearrangements and remodeling of higher-order protein assemblies. We therefore prioritized reactive cysteines that were highly conserved^54^, predicted to be pathogenic by AlphaMissense, located proximal to known disease variants (ClinVar^59^), and mapped to proteins within known complexes^55, 56, 57, 58^ (Fig. 4a).

This analysis identified 32 differentially reactive cysteines in 26 high-confidence protein targets, including PPP1R21, also known as Fy2, a subunit of the recently described FERRY complex^20, 21^ (Fig. 4a-d, Extended Data Fig. 6c). The FERRY complex is a five-membered Rab5 effector originally characterized in neurons, where it associates with early endosomes to facilitate mRNA transport, subcellular localization, and localized translation^20, 21^ (Fig. 4b). More recently, Fy2 has been proposed to form a three-subunit complex with Fy4 and Fy5 that mediates mRNA transport more broadly across the endo-lysosomal system^60, 61^. Three of the five FERRY subunits detected in our TMT-exp dataset, Fy1, Fy2, and Fy4, showed coordinated post-transcriptional upregulation of protein expression following macrophage activation (Fig. 4e).

TMT-ABPP revealed stimulation-dependent reactivity changes of both Fy1_C737 and Fy2_C696 (Fig. 4c-e). Fy2 serves as the central scaffold of the FERRY complex through interactions with mRNA cargo, Fy1, Fy3, Fy4, Fy5, and GTP-bound Rab5^20^. Notably, the differentially reactive Fy2_C696 lies in the established Fy1/Fy3 interaction interface at the *C*-terminal domain (residues 646–705)^20^, suggesting that its increased reactivity may reflect stimulation-induced remodeling of the FERRY complex. While structural information is currently unavailable for Fy1, C737 lies on the same tryptic peptide as a reported phosphotyrosine pY732^62^, raising the possibility of post-translational regulation.

While the FERRY complex has primarily been studied in neurons, where it mediates mRNA transport required for axonal function, localized translation in macrophages has been reported for only a limited number of transcripts, including actin mRNA at phagocytic cups^63^. To determine whether the complex is assembled in macrophages, we performed IF analysis of Fy1, Fy2, Fy4, and Rab5 in resting and activated cells (Fig. 4f-h, Extended Data Fig. 6d,e; Data S4). All FERRY subunits displayed punctate localization consistent with membrane-associated compartments. Rab5 and Fy2 showed approximately 50% colocalization, consistent with the reported localization of Fy2 to early endosomes. Fy4 and Fy2 also showed approximately 50% colocalization (Extended Data Fig. 6d,e). In contrast, Fy1 showed only approximately 20% colocalization with Fy2 under basal conditions, consistent with the established functions of Fy1 in other signaling pathways^60^. The colocalization of Fy2 with Rab5 remained unchanged following TLR4 stimulation, suggesting that these proteins are constitutive interactors in our model. By comparison, colocalization of both Fy1 and Fy4 with Fy2 increased moderately following TLR4 stimulation (Fig. 4h and Extended Data Fig. 6e). These results support a model in which Fy2 is constitutively associated with Rab5, while recruitment of other complex members may be context-dependent. This interpretation is consistent with previous evidence for state-dependent recruitment of Fy1 and Fy3 to a stable three-membered complex between Fy2, Fy4, and Fy5^61^.

### Orthogonal profiling defines the FERRY interactome and identifies DISC1 as a macrophage Fy2-interacting protein

To validate state-dependent FERRY complex assembly and identify macrophage-specific interacting proteins, we performed complementary immunoprecipitation-mass spectrometry (IP-MS) and imaging analyses (Fig. 5a). IP-MS of Fy1 and Fy2 confirmed enrichment of core FERRY subunits in both M0 and TLR4-stimulated macrophages (Fig. 5b,c and Extended Data Fig. 7a-d; Data S2–7), with the exception of Fy5, which was detected only in Fy2 pulldowns (Extended Data Fig. 7b-c). Fy2 IP-MS showed increased enrichment of Fy1 and Fy3 following TLR4 stimulation (Fig. 5c), consistent with the IF colocalization data and the proposed state-dependent recruitment of these complex members. Reciprocally, Fy1 IP-MS showed increased enrichment of Fy2 and Fy4 in TLR4-stimulated macrophages, providing further support for this model (Extended Data Fig. 7d).

The Fy1 interactome was substantially broader than that of Fy2, suggesting that Fy1 may have functions beyond its role in the FERRY complex, potentially mediated by its TBC domain which is characteristic of RabGAP proteins^66^. Consistent with this, gene ontology analysis of molecular functions enriched among Fy1-interacting proteins identified cytoskeletal binding, motor activity, and GTPase binding (Extended Data Fig. 7e). Considering the breadth of the Fy1 interactome and the central scaffolding role of Fy2 within the FERRY complex, we focused subsequent analyses on Fy2-associated proteins.

We next compared our combined macrophage Fy2 interactome with FERRY interactomes generated by GST-FERRY IP-MS from HEK293 cell lysates^21^ and Fy1/2 affinity purification mass spectrometry (AP-MS) in HeLa cells^60^. All five core FERRY subunits were consistently enriched across all datasets (Extended Data Fig. 7f,g). This comparison also identified candidate macrophage-specific interacting proteins, including the scaffolding protein DISC1, which associated with Fy2 in both resting and TLR4-stimulated macrophages but was not detected previously. We confirmed enrichment of core complex members and DISC1 by Western blot (Fig. 5d). Finally, to assess FERRY complex assembly in intact cells, we performed proximity-ligation assays (PLA), which report physical proximity of two proteins as discrete fluorescent puncta (Fig. 5e and Extended Data Fig. 7h,i). Analysis of PLA results supported the proximity of Fy2 to Rab5, Fy1, Fy4, and DISC1 in primary human macrophages, further validating FERRY complex assembly and its association with DISC1.

Combined, our findings support a model in which FERRY assembles and associates with early endosomes in primary human macrophages, with TLR4 stimulation promoting post-translational recruitment of Fy1 and Fy3 to the complex (Fig. 5f). We further identify DISC1 as a previously unrecognized Fy2-interacting protein. Future studies will define the role of DISC1 in FERRY complex function and determine how remodeling of the complex contributes to macrophage activation.

## DISCUSSION

Macrophages are central effectors of innate immunity and contribute to the pathogenesis of inflammatory and autoimmune diseases and cancer. Activation of cell-surface and endosomal TLRs, as well as cytosolic DNA-sensing cGAS-STING pathway, induces inflammatory programs that have been extensively characterized at the transcriptional level. However, macrophage activation also involves rapid post-translational regulation that governs protein abundance, activity, localization, and molecular interactions to remodel metabolism, organelle function, and membrane trafficking. The scope of this regulatory layer remains incompletely defined, in part because conventional proteomic approaches do not capture the full spectrum of biochemical changes associated with macrophage activation that occur independently of RNA or protein abundance changes.

Here, we integrated transcriptomics, global proteomics, phosphoproteomics, and PTM-agnostic, multiplexed cysteine reactivity profiling by TMT-ABPP to map the post-translational landscape of primary human macrophages following TLR and STING activation. These complementary approaches revealed substantial discordance between transcript- and protein-abundance changes, as well as extensive post-translational remodeling not captured by abundance measurements alone. Proteins exhibiting abundance changes in the absence of corresponding transcriptional regulation were enriched in metabolic pathways, mitochondrial gene expression, and mitochondrial translation – processes that support macrophage activation and require rapid remodeling in response to extracellular cues. Our analyses further identified differential protein turnover as a potential contributor to the observed divergence between transcript- and protein-level changes, although observed discordance could also be due to other factors such as protein translation rates and stabilizing interactions. These findings highlight the contributions of post-transcriptional mechanisms to proteome remodeling during macrophage activation.

Cysteine reactivity profiling provided an orthogonal readout of changes in native protein states. Cysteine labeling by electrophilic probes can be influenced by local structure, ligand occupancy, protein interactions, subcellular environment, and reversible or irreversible covalent modifications. Accordingly, differential reactivity does not by itself identify a specific regulatory mechanism, but it can nominate protein sites undergoing biochemical remodeling that would not be detected by conventional unenriched proteomics or PTM-focused analyses. Reactivity changes observed following macrophage activation showed limited overlap with those identified in our previous studies of T cell activation^15^ and dysfunction^18^. Although differences in protein coverage and stimulation conditions may contribute to this limited overlap, the results suggest that cysteine reactivity profiling can capture distinct biochemical states across immune cell populations.

Cysteine reactivity profiling revealed extensive remodeling of small-GTPase signaling and vesicular trafficking pathways, including endosomal transport and autophagy. These processes are central to macrophage biology, controlling receptor trafficking, phagocytosis, cytokine secretion, antigen processing, and organelle homeostasis. Among these changes, we identified altered cysteine reactivity in the nucleotide-binding pocket of CDC42, an important GTPase with context-dependent functions in macrophages^67, 68, 69, 70^ regulating actin remodeling, cell migration, phagocytosis, and inflammatory signaling. TLR4 stimulation has been reported to activate CDC42 in macrophage models^71^. In lysate-based metabolite-addback experiments, GTP, but not ATP, decreased CDC42_C18 labeling by a cysteine-reactive maleimide probe, with a more pronounced effect in basal state M0 macrophages compared to TLR4-stimulated cells. Whole-cell GTP abundance did not change significantly following macrophage activation, indicating that differential CDC42_C18 reactivity is unlikely to be explained by changes in bulk intracellular GTP concentration, but may instead reflect targeted regulation of CDC42 by GEFs and GAPs that affect nucleotide occupancy, protein conformation, or effector protein interactions. It is also worth noting that CDC42_C18 is located within a redox-sensitive motif conserved among several Rho-family GTPases, raising the possibility that reactivity could also be influenced by oxidative modifications, including *S-*nitrosylation or sulfenylation^72, 73, 74^. While the current data do not distinguish among these mechanisms, it nevertheless illustrates how cysteine reactivity profiling can detect regulated changes at functional protein sites that are not apparent from metabolite or protein abundance measurements alone.

Integrated analysis of structural, evolutionary, and disease-associated annotations further prioritized cysteines in the FERRY complex as candidate sites of biochemical or post-translational regulation in primary human macrophages. FERRY is a Rab5 effector that associates with early endosomes and select mRNAs, enabling endosome-dependent mRNA transport and localized translation. We identified activation-associated reactivity changes in two subunits of this five-membered complex: the central scaffold Fy2 (PPP1R21) and its direct interactor Fy1 (TBCK)^21^. While the FERRY complex has been characterized in neurons and other cell lines, its assembly and function in immune cells has remained largely unknown. We validated complex assembly and localization to early endosomes in primary human macrophages using complementary biochemical, proteomic, and imaging approaches, including IP-MS of both Fy1 and Fy2, immunofluorescence, and proximity ligation assays. These findings extend FERRY beyond the systems in which it was originally characterized and raise the possibility that endosome-associated mRNA transport contributes to innate immune cell function.

Our IP-MS analysis further identified DISC1 as a previously unrecognized Fy2 interactor, which we validated by Western blot analysis following Fy2 immunoprecipitation, and by PLA. DISC1 is a multifunctional scaffolding protein that coordinates microtubule-dependent transport, including mRNA and mitochondria trafficking^75^, but has not been previously linked to FERRY. FERRY preferentially associates with subsets of transcripts, including mRNAs encoding mitochondrial proteins, and mRNA-loaded FERRY-positive endosomes have been observed in close proximity to mitochondria^20, 21^. Recent work has also supported a potential role of the dynein-dynactin adaptor JIP4 in the endo-lysosomal FERRY trafficking^61^. In this context, the identified Fy2-DISC1 association suggests that DISC1 may help coordinate the movement or positioning of FERRY-positive endosomes relative to mitochondria, potentially promoting spatially restricted delivery or translation of mitochondrial transcripts. Further studies will be required to define the molecular basis and functional significance of this interaction during macrophage activation.

Localized translation remains poorly understood in immune cells. During innate immune activation, macrophages remodel their endosomal and lysosomal compartments, cytoskeleton, secretory pathways, and metabolic state. Coupling select transcripts to motile endosomes could allow protein synthesis to be coordinated with these spatially restricted changes. Our findings raise the possibility that FERRY participates in this process, but its RNA cargoes and functional outputs in macrophages remain unknown. Identifying FERRY-associated transcripts in resting and activated macrophages, determining whether they are translated near endosomes or other organelles, and perturbing individual FERRY subunits will be necessary to establish whether this pathway regulates inflammatory signaling, organelle homeostasis, or other macrophage functions.

Several limitations should be considered. Our study focused on a 24-hour endpoint and therefore doesn’t capture the dynamic regulatory events that occur during earlier stages of macrophage activation. Moreover, cysteine reactivity changes can arise through multiple mechanisms and require site-specific biochemical validation before they can be assigned to changes in protein activity or a particular post-translational modification. Similarly, metabolite addback experiments performed in lysates do not preserve subcellular compartmentalization of metabolite pools and regulatory layers present in intact cells. Finally, our FERRY studies establish complex assembly, localization, and its association with DISC1 in macrophages but do not yet define its RNA cargoes or functional contribution to macrophage activation. Addressing these questions will require additional investigations that extend beyond the current study.

In summary, our integrated multi-omic analysis provides a comprehensive view of biochemical and post-translational changes accompanying TLR and STING activation in primary human macrophages. The data identify extensive remodeling of small-GTPase signaling and the endo-lysosomal system, nominate functional cysteines that may report on changes in nucleotide occupancy and other regulatory states, and establish the presence and assembly of the FERRY mRNA transport complex in macrophages. These datasets provide a rich resource for identifying regulatory nodes of macrophage activation and lay the groundwork for future investigations into the role of mRNA transport and localized translation in innate immunity.

## Extended Data

**Extended Data Fig. 1.**
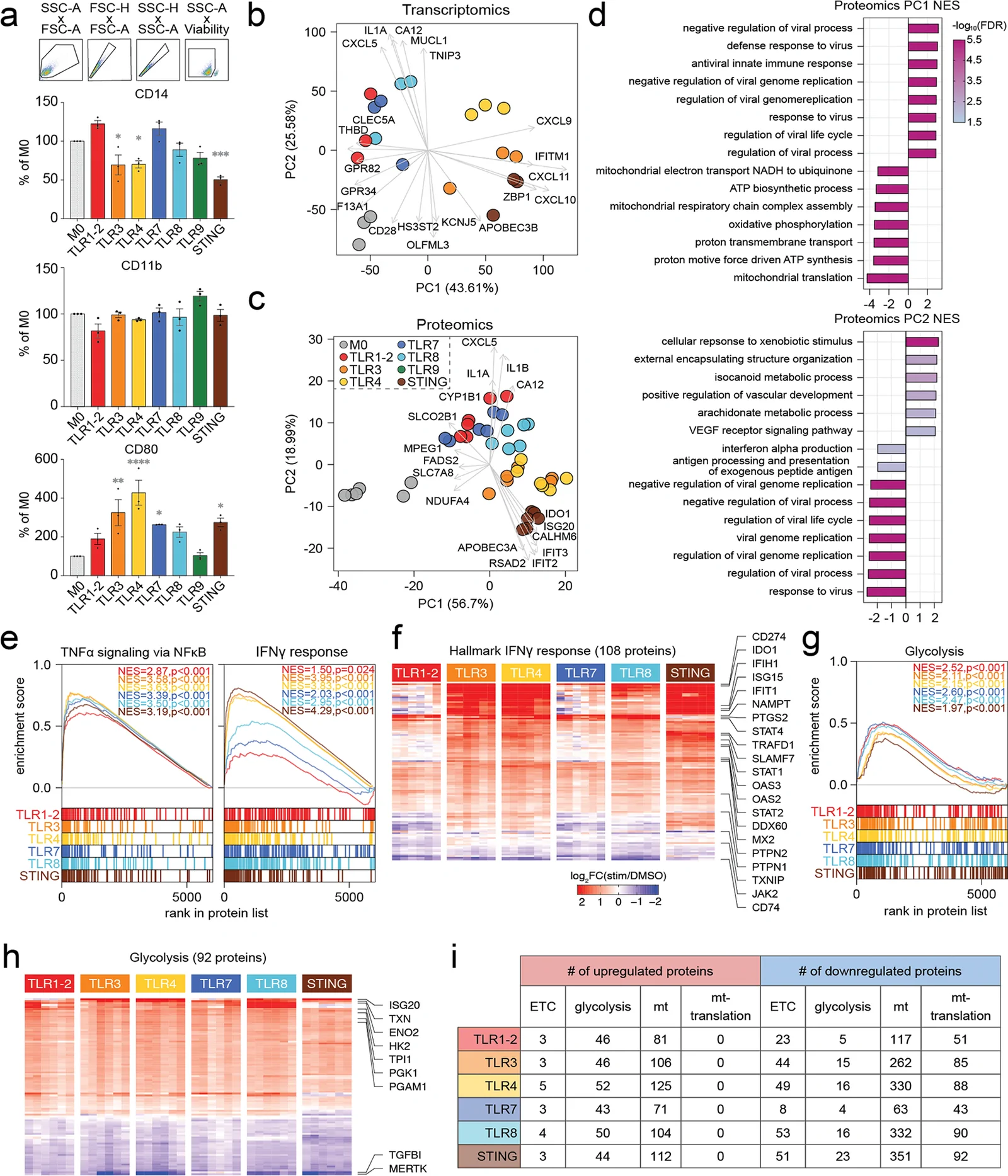
Multi-omic profiling of TLR- and STING-stimulated macrophages reveals induction of pro-inflammatory pathways (Related to Figure 1). (**a**) Representative flow cytometry gating strategy (top) and quantification of cell-surface expression of CD14, CD11b, and CD80 across macrophage activation states (bottom). Data are presented as the average ± SEM of three donors. Statistical significance was determined by one-way matched ANOVA with Dunnett’s multiple comparisons test (ns, p > 0.05; *, p < 0.05; **, p < 0.01; ***, p < 0.001; ****, p < 0.0001). (**b**) Principal component analysis (PCA) of bulk transcriptomic data from n = 3 donors. Normalized count values were log_2_ transformed, and the 5,000 protein-coding genes with the highest cross-experiment variability were used. The five highest and five lowest loadings from PC1 and PC2 were plotted. (**c**) PCA of unenriched proteomic data from n = 3 donors. Protein abundance ratios were log_2_ transformed and only proteins quantified in all experiments were used. The five highest and five lowest loadings from PC1 and PC2 were plotted. (**d**) Gene set enrichment analysis (GSEA) of PCA loading scores using the Gene Ontology Biological Process database. Top 15 gene sets based on normalized enrichment scores (NES) are plotted for PC1 (top) and PC2 (bottom). (**e**) GSEA of the Hallmark TNFα signaling via NFκB and IFNγ response gene sets. Each stimulation condition was compared to unstimulated M0 macrophages. NES, normalized enrichment score. (**f**) Heatmap displaying abundance changes for proteins in the Hallmark IFNγ response gene set relative to M0 cells. Proteins were hierarchically clustered. (**g**) GSEA of the Hallmark Glycolysis list. Each stimulation condition was compared to M0 cells. NES, normalized enrichment score. (**h**) Heatmap displaying abundance changes for proteins in the Hallmark Glycolysis gene set relative to M0 cells. Proteins were hierarchically clustered. (**i**) Numbers of upregulated and downregulated proteins in each macrophage activation state associated with GO terms ETC, Glycolysis, Mitochondria, and Mitochondrial translation.

**Extended Data Fig. 2.**
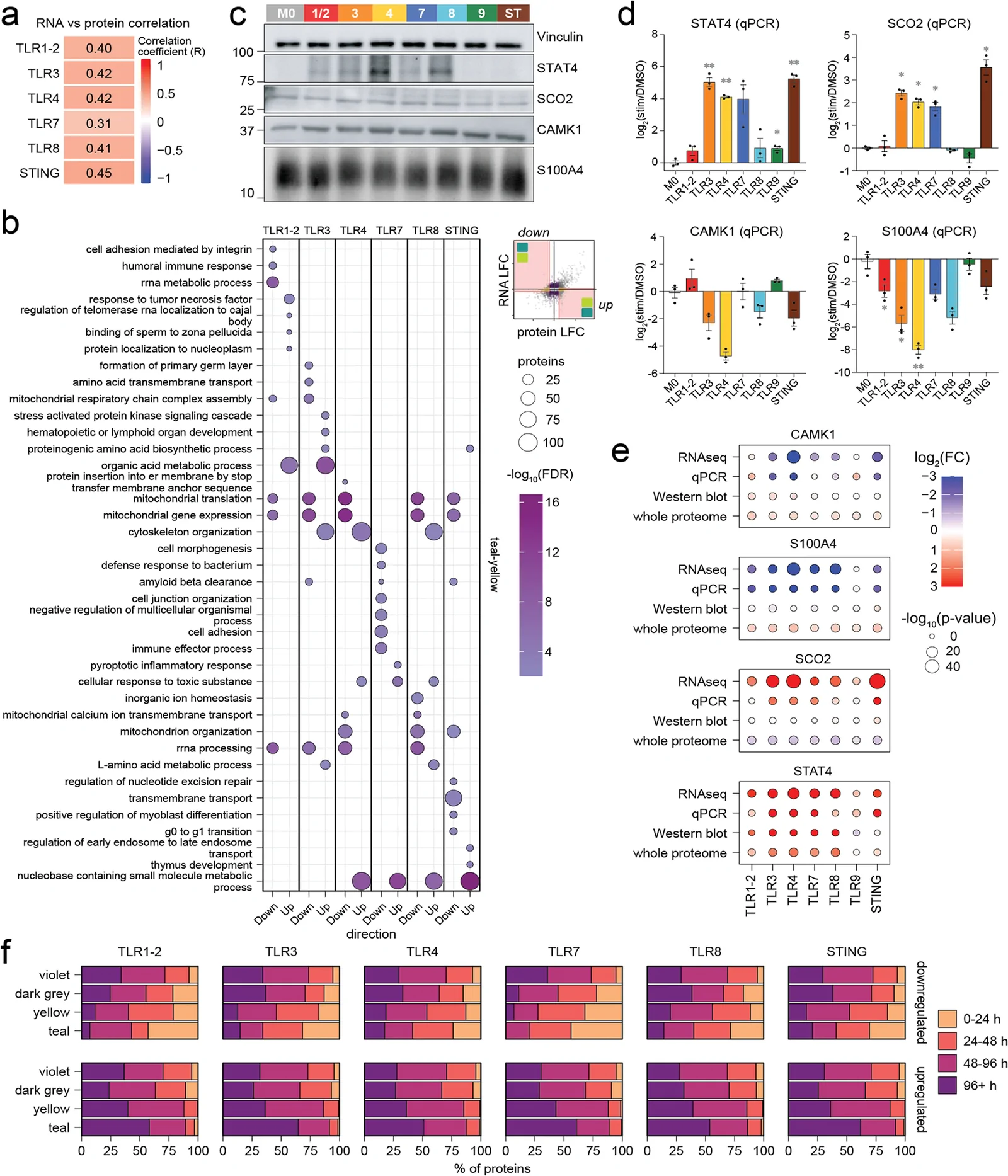
Characterization of genes with discordant protein- and transcript-level changes (Related to Figure 1). (**a**) Heatmap of Pearson’s Correlation Coefficients comparing protein- and transcript-level log_2_ fold changes (LFC) across macrophage stimulation conditions compared to unstimulated M0 cells. (**b**) Overrepresentation analysis (Fisher’s exact test, experiment-specific background) of proteins changing in abundance without corresponding transcriptional changes across macrophage stimulation conditions compared to M0 cells (yellow and teal quadrants in Fig. 1h). Terms with FDR < 0.01 and protein number > 5 are shown. Reg., regulation; Up, positive protein LFC; Down, negative protein LFC. (**c**) Representative Western blots of selected proteins showing concordant or discordant protein- and transcript-level changes following macrophage stimulation. (**d**) RT-qPCR quantification of transcript levels for selected genes. Data shown represents average ± SEM from three donors, and statistical comparison is by one-way matched ANOVA with Dunnett’s multiple comparisons test (ns, p > 0.05; *, p < 0.05; **, p < 0.01). (**e**) Summary of transcript-level (RNA-seq and RT-qPCR) and protein-level (Western blot and abundance-based proteomics) changes in STAT4, SCO2, CAMK1, and S100A4 across macrophage activation conditions compared to M0 cells. p-values were calculated depending on data modality: DESeq2 for RNA-Seq, ANOVA for RT-qPCR, one sample t-test (against 1, the value M0 is normalized to) for Western blotting, and unpaired t-test for TMT-exp (consistent throughout the manuscript). Fold-change values represent the mean for each modality. (**f**) Reference protein half-lives in monocytes^1^ for upregulated and downregulated proteins, depending on their correlation with transcript-level changes. Half-life values represent the mean of two biological replicates, and only proteins with reported half-life data are included.

**Extended Data Fig. 3.**
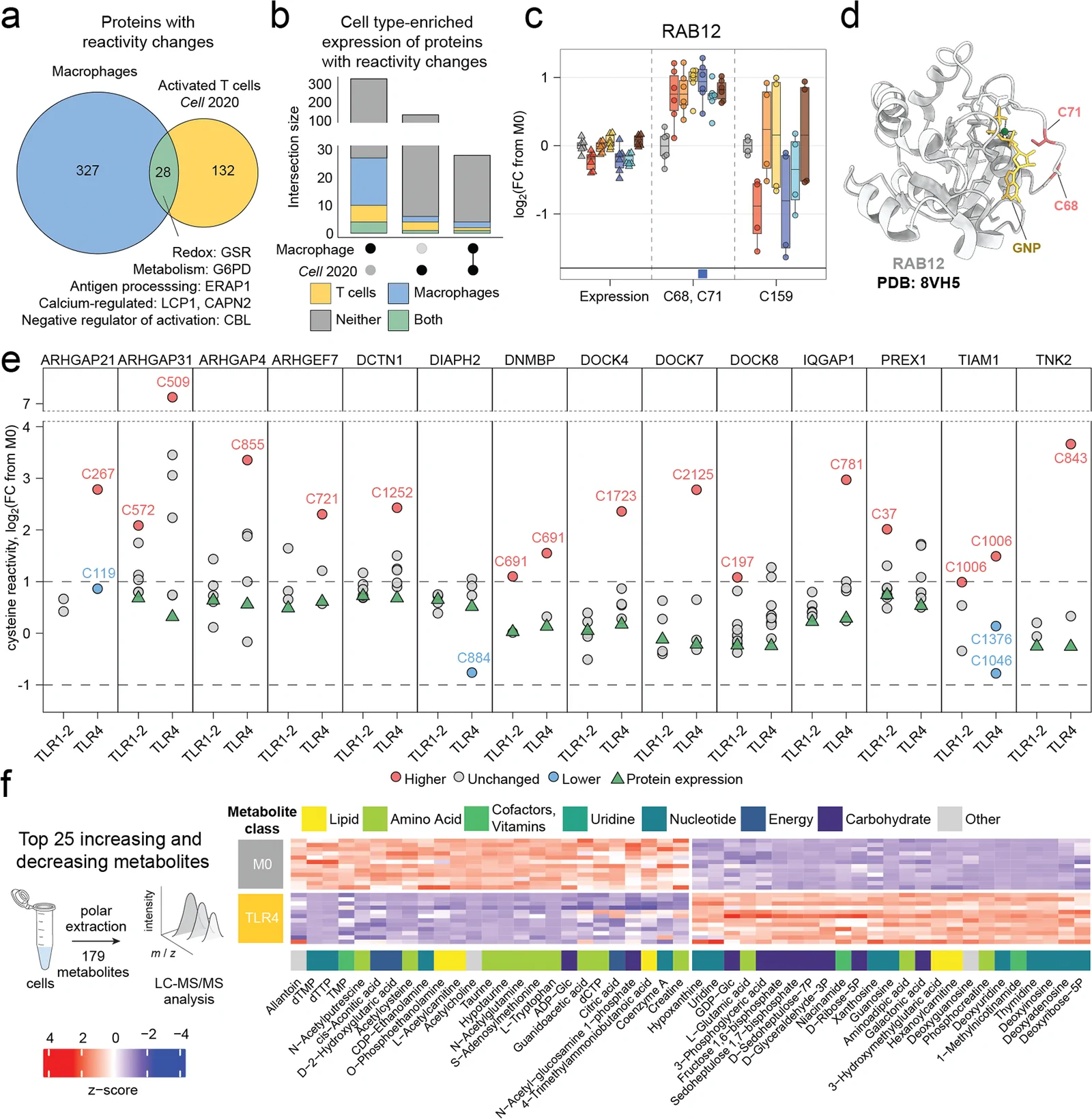
Cysteine reactivity profiling and metabolomics reveal remodeling of GTPases and their regulatory networks (Related to Figure 2). (**a**) Venn diagram comparing proteins with reactivity changes identified in TLR- and STING-stimulated primary human macrophages (this study; blue) with those identified in TCR-stimulated primary T cells^2^. Select proteins in the intersection are highlighted. (**b**) UpSet plot comparing proteins with cysteine reactivity changes in primary human macrophages and T cells^2^. Bars are colored according to cell type-enriched expression annotations from the Human Protein Atlas^3^, combining the “Cell type enhanced,” “Cell type enriched,” and “Group enriched” categories. (**c**) Box plots showing changes in RAB12 expression (triangles) and cysteine reactivity (dots) across macrophage stimulation conditions compared to M0 cells. Boxes indicate the 25th to 75th percentiles, and whiskers extend to 1.5x the interquartile range. Boxes on the bottom margin indicate cysteines classified as differentially reactive. (**d**) Cryo-EM structure of RAB12 bound to a GTP analog (PDB 8VH5^4^). Cysteines contained within the same differentially reactive tryptic peptide are highlighted in red. (**e**) Log_2_ fold changes in protein expression and cysteine reactivity for CDC42-interacting proteins in TLR1/2- and TLR4-stimulated macrophages relative to M0 cells. Protein expression changes are shown as green triangles and cysteine reactivity changes as dots. Cysteine reactivity and protein expression data are from n = 3 donors. (**f**) Heatmap showing z-scores calculated from metabolite signal intensity in M0 and TLR4-stimulated macrophages. Top 25 increasing and decreasing metabolites by p-value between TLR4-stimulated macrophages (bottom) and M0 cells (top) are shown. Annotations at the bottom indicate metabolite class. Data are from n = 4 donors.

**Extended Data Fig. 4.**
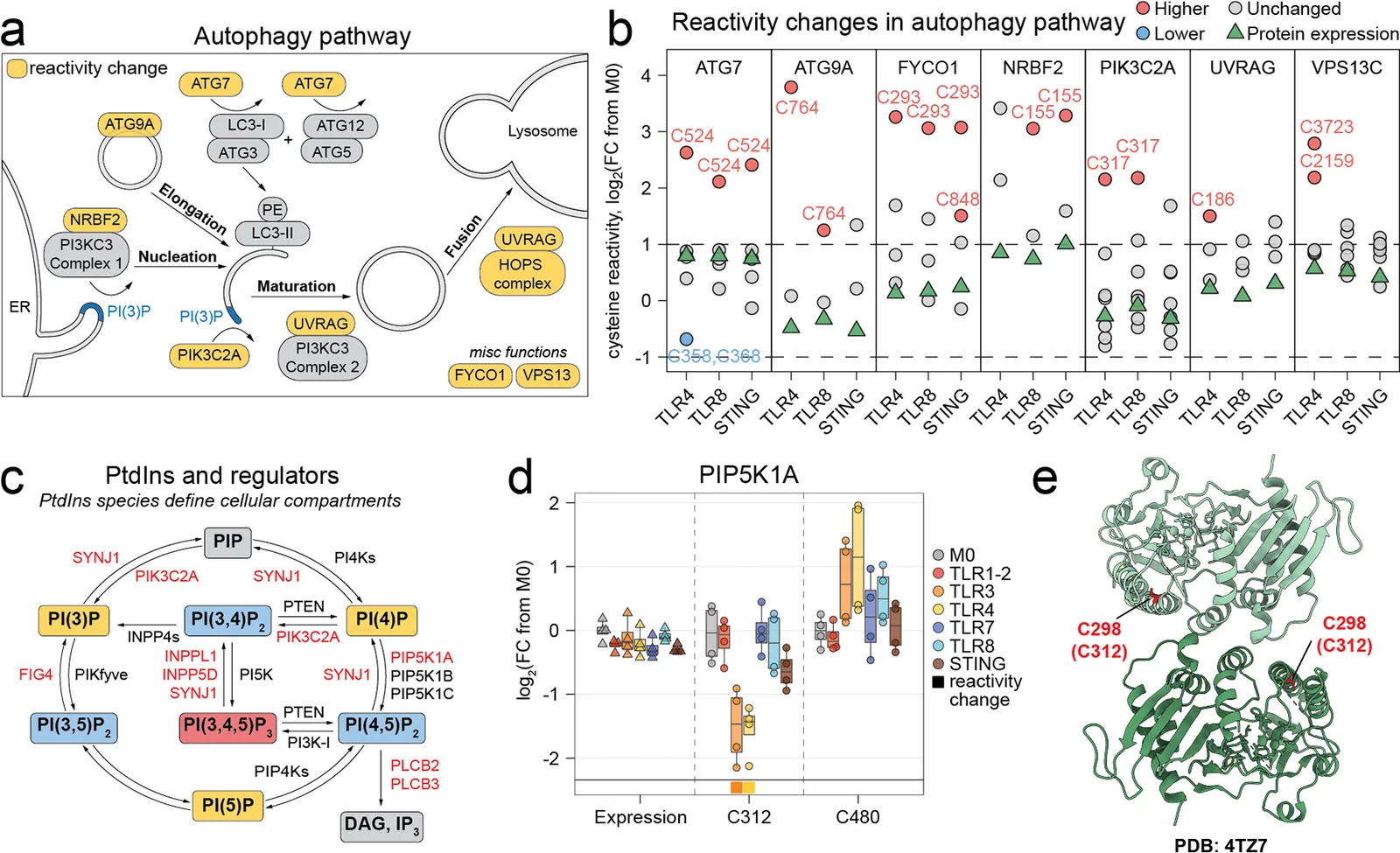
Cysteine reactivity profiling reveals remodeling of autophagy pathways and phosphoinositide regulators (Related to Figure 3). (**a**) Schematic of the autophagy pathway. Proteins with reactivity changes in any condition are highlighted in yellow. (**b**) Log_2_ fold changes in protein expression and cysteine reactivity for proteins in the autophagy pathway in TLR4-, TLR8-, and STING-stimulated macrophages relative to M0 cells. Protein expression changes are shown as green triangles and cysteine reactivity changes as dots. Cysteine reactivity and protein expression data are from n = 3 donors. (**c**) Schematic of phosphoinositide species and the lipid kinases and phosphatases that catalyze their interconversion. PtdIns containing one, two or three phospho groups are shown in yellow, blue, and red, respectively. Proteins with cysteine reactivity changes are highlighted in red. (**d**) Box plots showing changes in PIP5K1A expression (triangles) and cysteine reactivity (dots) across macrophage stimulation conditions compared to M0 cells. Boxes indicate the 25th to 75th percentiles, and whiskers extend to 1.5x the interquartile range. Boxes on the bottom margin indicate cysteines classified as differentially reactive. (**e**) Crystal structure of the zebrafish PIP5K1A dimer (PDB 4TZ7^5^), with the cysteine corresponding to the differentially reactive C312 in human PIP5K1A (C298 in zebrafish) highlighted.

**Extended Data Fig. 5.**
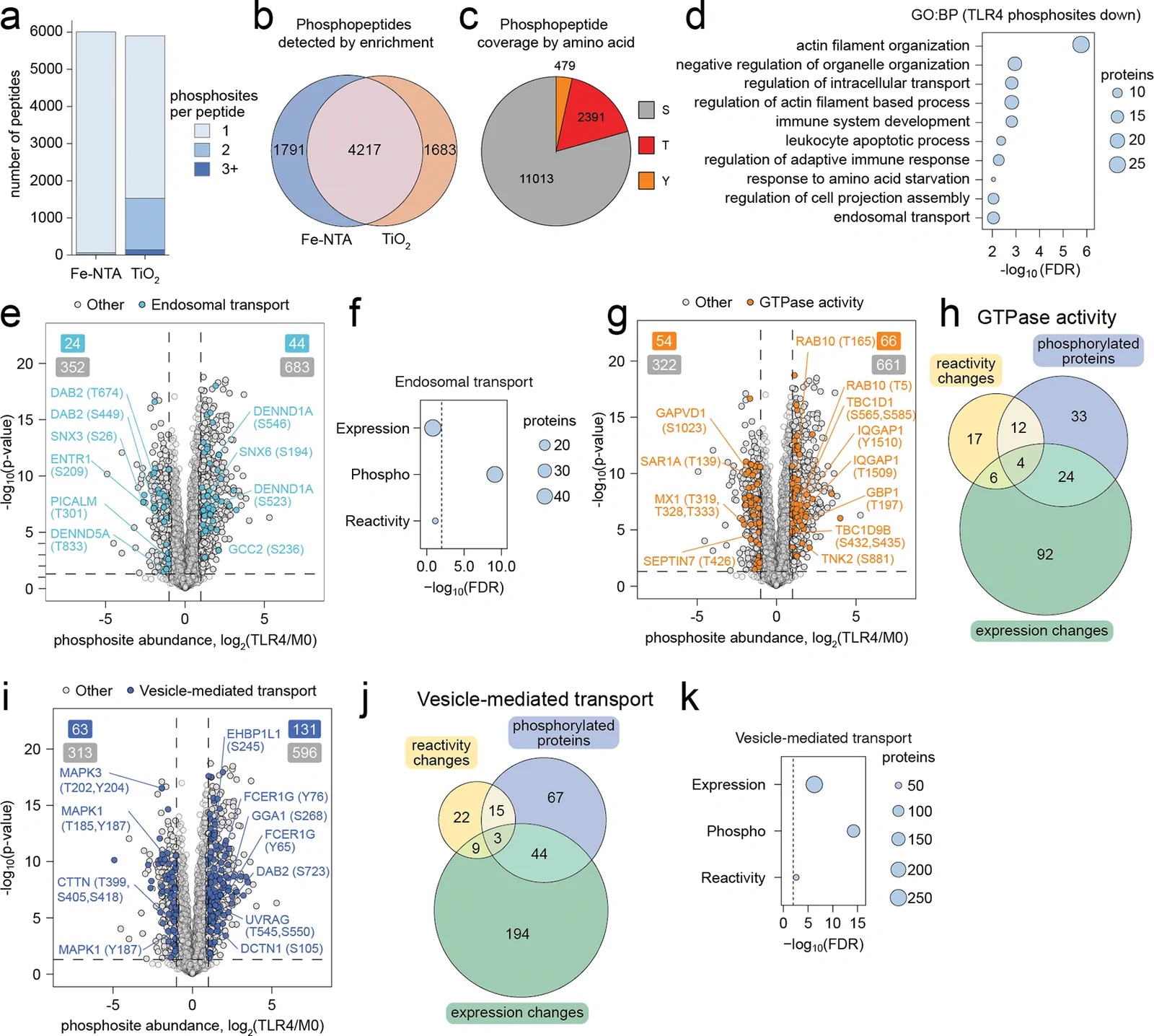
Phosphoproteomic profiling reveals enrichment of vesicle trafficking pathways in TLR4-stimulated macrophages (Related to Figure 3). (**a**) Phosphopeptides detected following Fe-NTA and TiO_2_ enrichment, colored by the number of phosphosites per peptide. Data are from n = 4 donors. (**b**) Venn diagram comparing phosphopeptides detected following Fe-NTA and TiO_2_ enrichment. (**c**) Pie chart showing the distribution of serine, threonine, and tyrosine phosphosites detected using the dual TiO_2_/Fe-NTA enrichment workflow. (**d**) GO:BP enrichment analysis of proteins containing phosphosites with significantly decreased phosphorylation in TLR4-stimulated cells compared to M0 (FC < 0.5, p < 0.05). Terms with FDR < 0.01 and protein number > 5 are shown. **(e)** Volcano plot showing log_2_ fold changes of phosphopeptides between TLR4-stimulated and unstimulated M0 cells where each point represents a phosphosite, with proteins annotated in the endosomal transport gene ontology term (GO:0016197) highlighted in teal. **(f)** Comparative enrichment of the endosomal transport gene set across expression proteomics, reactivity profiling, and phosphoproteomic datasets. **(g)** Volcano plot showing log_2_ fold changes of phosphopeptides between TLR4-stimulated and unstimulated M0 cells where each point represents a phosphosite, with proteins annotated in the GTPase activity gene ontology term (union of molecular function terms related to GTPase activity, activation, regulation, inhibition, and binding) highlighted in orange. **(h)** Venn diagram of the differentially regulated proteins identified by expression proteomics, cysteine reactivity profiling, and phosphoproteomic datasets within the GTPase activity gene set. **(i)** Volcano plot showing log_2_ fold changes of phosphopeptides between TLR4-stimulated and unstimulated M0 cells where each point represents a phosphosite, with proteins annotated in the vesicle-mediated transport gene ontology term (GO:0016192) highlighted in blue. **(j)** Venn diagram of the differentially regulated proteins identified by expression proteomics, cysteine reactivity profiling, and phosphoproteomic datasets within the vesicle-mediated transport gene set. (**k**) Comparative enrichment of the vesicle-mediated transport gene set across expression proteomics, reactivity profiling, and phosphoproteomic datasets. Gene sets for e-k were tested independently against the TLR4-regulated proteins identified by cysteine reactivity profiling (FC > 2 relative to expression value), expression-based proteomics (upregulated, FC > 1.5, p < 0.05), or phosphoproteomics (upregulated, FC > 2, p < 0.05) in TLR4-stimulated macrophages compared to M0 cells. Enrichment was computed with Fisher’s exact test against all annotated genes as background, and p-values were adjusted using the Benjamini-Hochberg method. The dashed line marks FDR = 0.01. Venn diagrams comparing proteins show changes in cysteine reactivity (FC > 2 relative to expression value), expression (FC > 1.5, p < 0.05), or phosphorylation (FC > 2, p < 0.05) in TLR4-stimulated macrophages compared to M0 cells.

**Extended Data Fig. 6.**
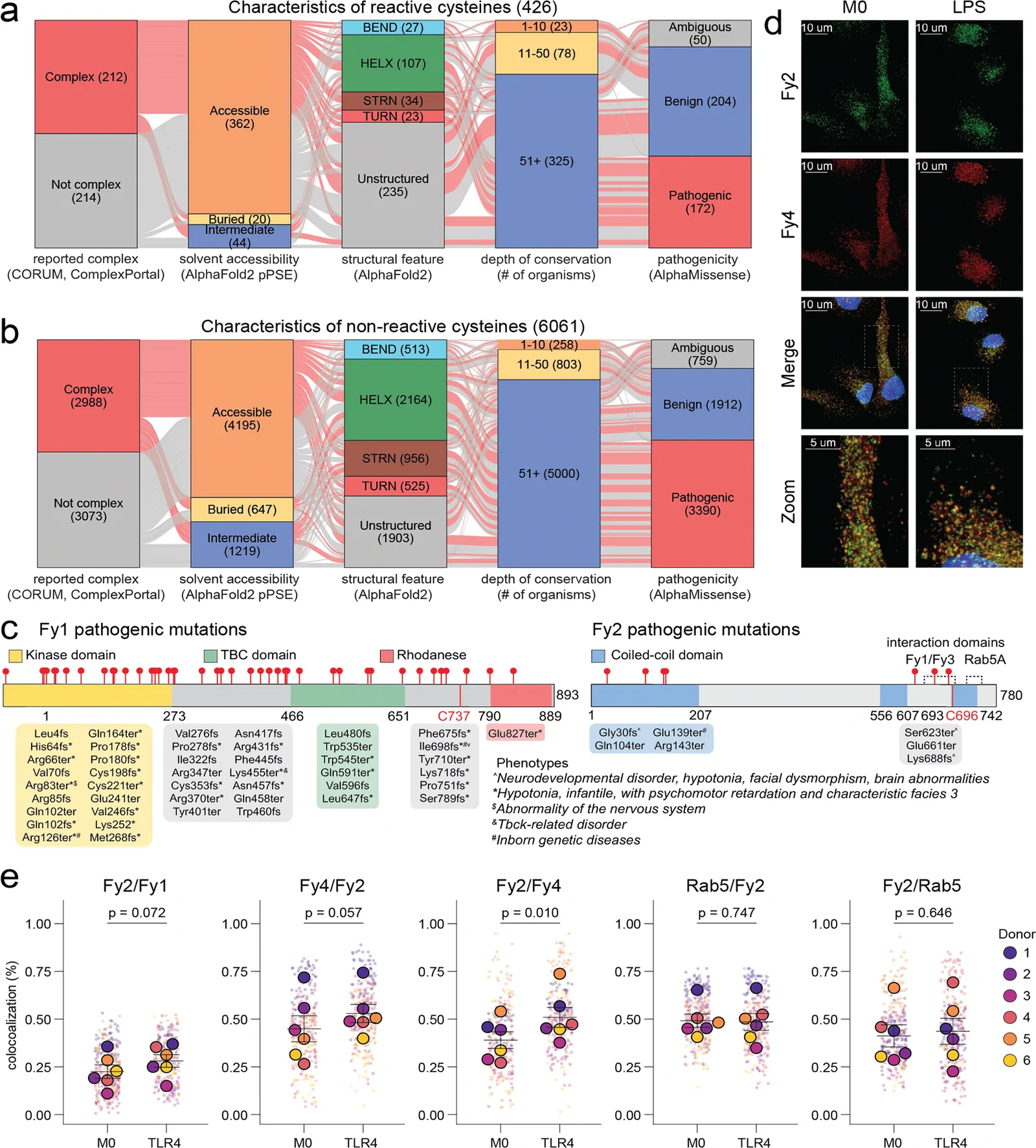
Prioritization of differentially reactive cysteines highlights assembly of the FERRY complex (Related to Figure 4). (**a, b**) Structural, evolutionary, and pathogenic features of differentially reactive cysteines compared with cysteines without reactivity changes. Alluvial diagrams partitioning (**a**) cysteines with significant reactivity changes (n = 399) and (**b**) cysteines without reactivity changes (n = 5,626) across five annotation categories: membership of the parent protein in a reported protein complex (CORUM) and Complex Portal; complex or not complex); predicted solvent accessibility based on AlphaFold2 structures (Buried = 9+ pPSE, Intermediate = 5–9 pPSE, Accessible = < 5 pPSE) local secondary structure (AlphaFold2/DSSP; helix [HELX], strand [STRN], turn [TURN], bend [BEND], or unstructured); evolutionary conservation, binned by the number of organisms in which the cysteine is conserved (1–10, 11–50, ≥51); and predicted variant pathogenicity (AlphaMissense classification: benign, ambiguous, or pathogenic). Each ribbon represents one cysteine. (**c**) ClinVar pathogenic or likely pathogenic mutations with a review status of at least one star mapped for Fy1 and Fy2. Variants are colored according to the protein domain they are in, with individual red markers denoting distinct variants. Unique markers denote corresponding phenotypes of each mutation. (**d**) Representative confocal microscopy images of Fy2 (red) and Fy4 (green) in M0 and TLR4-stimulated macrophages. White boxes highlight zoomed in regions. (**e**) Quantification of colocalized intensity between Fy1 and Fy2, Fy4 and Fy2, or Rab5 and Fy2. Average of cells per donor is represented by larger dot. Lines represent mean and SEM for n = 6 donors. Significance was determined by a two-tailed paired t-test on the donor means.

**Extended Data Fig. 7.**
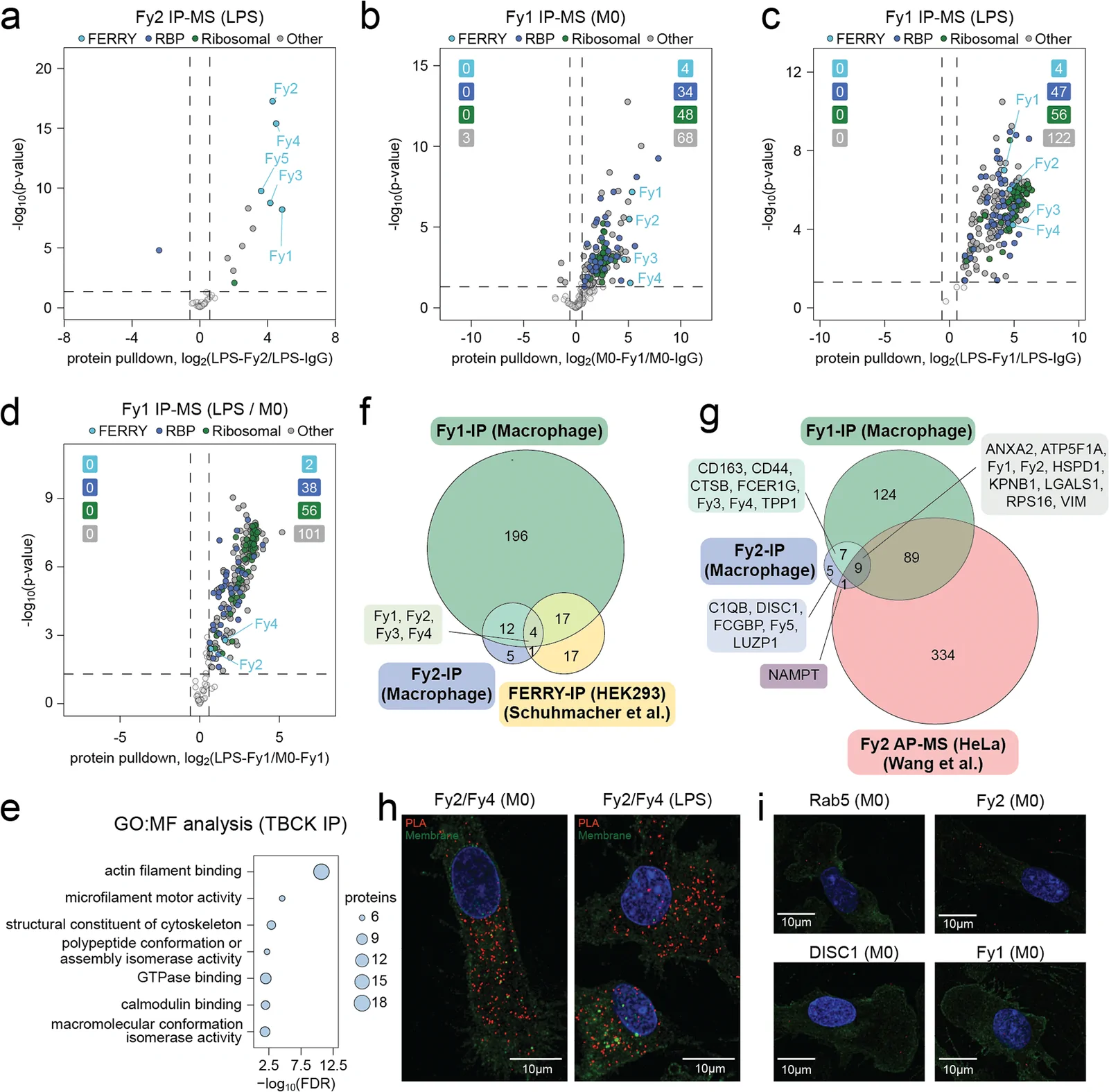
IP-MS and PLA reveal constitutive assembly of the FERRY complex, state-dependent recruitment of Fy1 and Fy3, and additional Fy1- and Fy2-interacting partners (Related to Figure 5). (**a-d**) Volcano plots of CRAPome-filtered^6^ proteins co-enriched with endogenous FERRY subunits Fy1 or Fy2 relative to IgG controls following immunoprecipitation from M0 and LPS-stimulated macrophage lysates (n = 3 donors). Vertical dashed lines mark the fold-change cutoff (±log_2_(1.5)) and the horizontal dashed line marks p = 0.05. Proteins exceeding both thresholds in the upper right quadrant are considered significantly enriched. Points are colored by protein category: FERRY complex subunits (light blue); RNA-binding proteins (RBP^7^; dark blue); ribosomal proteins (GO annotation; green); and all other proteins (grey). (**a**) Fy2 (PPP1R21) immunoprecipitation from LPS-stimulated macrophages, shown as log_2_(LPS-Fy2/LPS-IgG). All five FERRY subunits (Fy1-Fy5) were significantly co-enriched. (**b**) Fy1 (TBCK) immunoprecipitation from resting M0 macrophages, shown as log_2_(M0-Fy1/M0-IgG). Colored boxes in the upper corners show the number of significantly enriched proteins in each category. (**c**) Fy1 (TBCK) immunoprecipitation from LPS-stimulated macrophages, shown as log_2_(LPS-Fy1/LPS-IgG). (**d**) Comparison of proteins co-enriched with Fy1 (TBCK) in LPS-stimulated versus resting macrophages, shown as log_2_(LPS-Fy1/M0-Fy1). (**e**) Gene Ontology molecular function (GO:MF) enrichment analysis of proteins significantly enriched in endogenous Fy1 (TBCK) immunoprecipitations relative to IgG controls from M0 and LPS-stimulated macrophages. Enrichment was determined using Fisher’s exact test on the CRAPome-filtered^6^ enriched protein set. Each dot represents a GO:MF term; terms with FDR < 0.05 and n > 5 proteins are shown. (**f**,**g**) Venn diagrams comparing proteins significantly enriched in endogenous Fy1 (TBCK vs IgG) and Fy2 (PPP1R21 vs IgG) immunoprecipitations from macrophages with (**f**) the published GST-FERRY interactome from HEK293 cells^8^ (proteins annotated as “FERRY-complex enriched”) and (**g**) the published Fy2 (PPP1R21) AP-MS interactome from HeLa cells (Wang et al^9^.; supplementary Table S12, filtered to high-confidence, >1 peptide and abundance > 5000). (**h**,**i**) Representative PLA images detecting interactions between Fy2 and Fy4 (**h**) and corresponding negative controls (**i**). PLA signals are shown in red, and cell membranes and nuclei are shown in green and blue, respectively.

## Supplementary Material

Supplement 1

## Figures and Tables

**Fig. 1.**
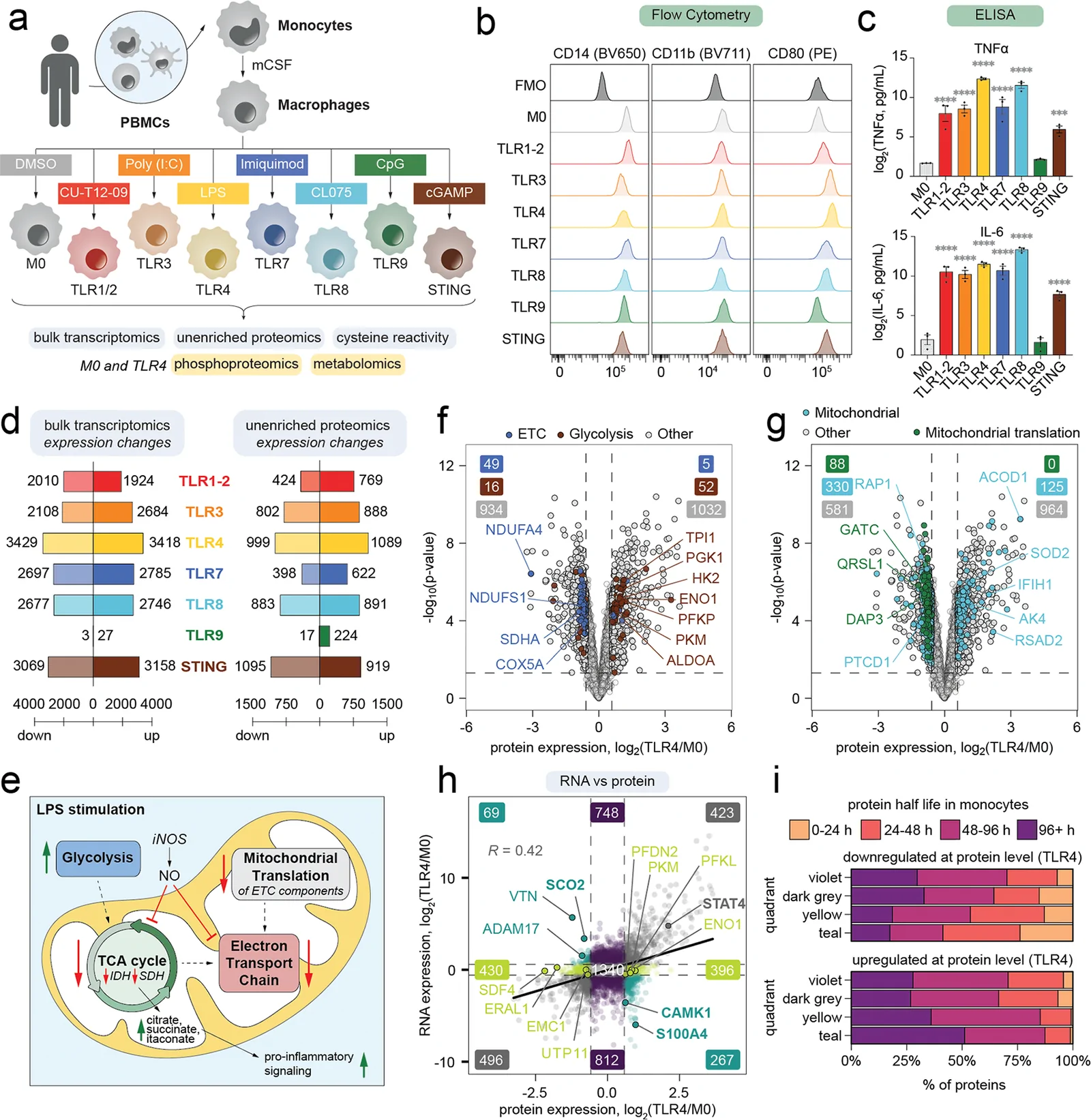
Transcriptomic and proteomic profiling of TLR- and STING-stimulated macrophages. **a**, Schematic of macrophage stimulation conditions and multi-omic platforms used to profile activated macrophages. **b**, Representative flow cytometry histograms for cell-surface expression of CD14, CD11b, and CD80 across macrophage activation states. **c**, Secreted TNF and IL-6 levels measured by ELISA in culture supernatants following 24 h of macrophage stimulation. **d**, Numbers of upregulated and downregulated transcripts and proteins identified by bulk RNA-seq (left; p < 0.05, FC > 2, n = 3 donors) and abundance-based proteomics (right; p < 0.05, FC > 1.5, n = 3 donors), respectively. **e**, Schematic illustrating metabolic rewiring in pro-inflammatory macrophages. **f**,**g**, Volcano plots showing log_2_(fold change) of proteins between TLR4-stimulated and unstimulated M0 cells. The horizontal dashed line marks p = 0.05 and the vertical dashed lines mark a 1.5-fold change. Proteins associated with glycolysis (Hallmark) and the electron transport chain (ETC; Gene Ontology) are highlighted according to their respective categories in (**f**), and proteins associated with mitochondria and mitochondrial translation are highlighted in (**g**). **h**, Scatter plot comparing log_2_(fold change) in protein abundance (x-axis) and transcript abundance (y-axis) between TLR4-stimulated and unstimulated M0 macrophages. Proteins are colored teal, yellow, purple or grey according to the concordance between protein- and transcript-level changes. Pearson’s correlation coefficient and regression line are shown. Only genes quantified in both proteomic and transcriptomic datasets are included. Transcriptomic and proteomic data are from n = 3 donors. **i**, Reference protein half-lives in monocytes^24^ for upregulated and downregulated proteins, depending on their correlation with transcript-level changes. Half-life values represent the mean of two biological replicates, and only proteins with reported half-life data are included.

**Fig. 2.**
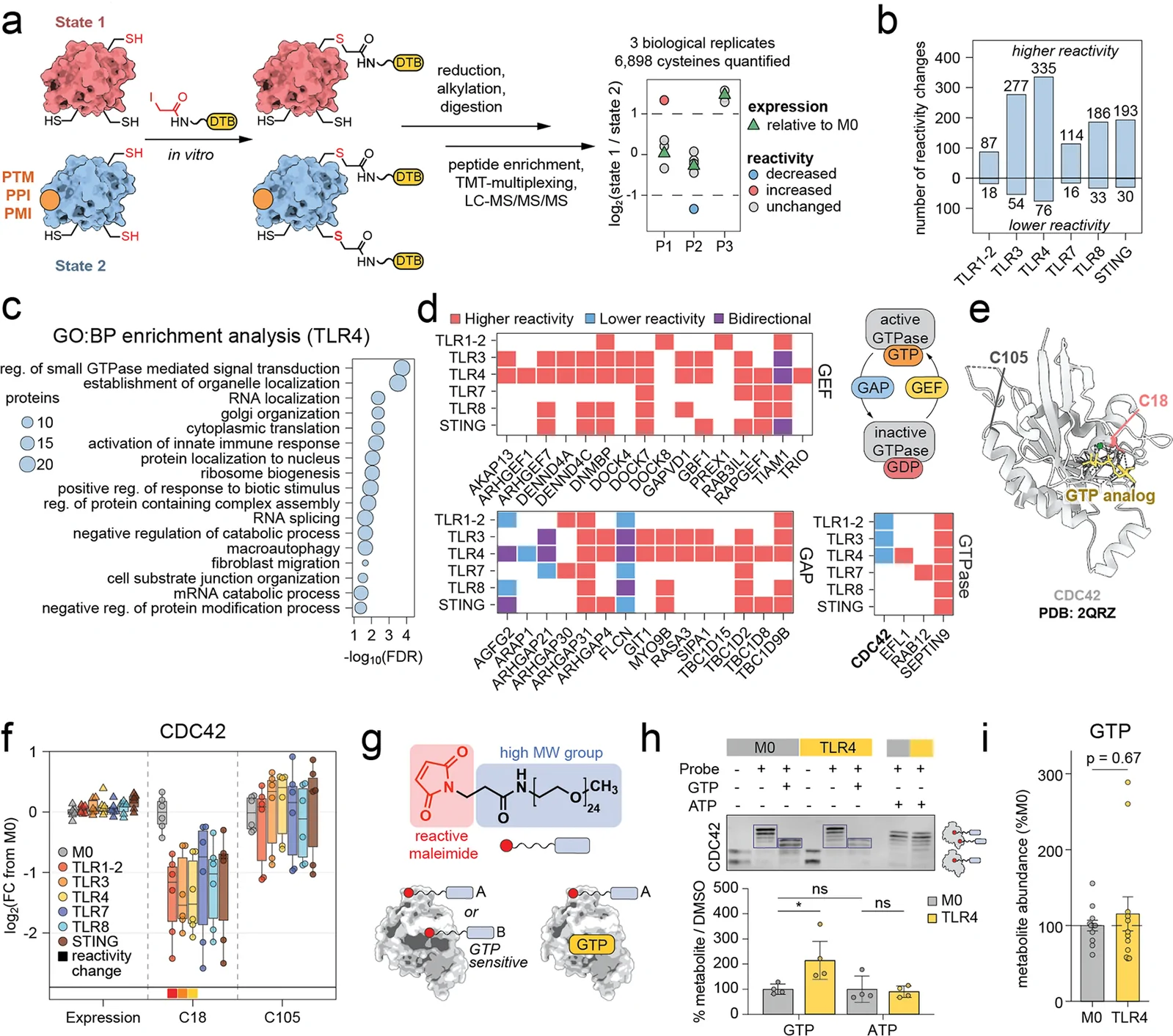
Cysteine reactivity profiling of TLR- and STING-stimulated macrophages reveals remodeling of GTPases and their regulators. **a**, Schematic of cysteine reactivity profiling and representative plot illustrating expression and reactivity changes. PTM, post-translational modification; PPI, protein-protein interaction; PMI, protein-metabolite interaction. **b**, Numbers of cysteines with reactivity changes across macrophage stimulation conditions relative to M0 cells (n = 3 donors). Differential reactivity was determined relative to changes in protein abundance measured by TMT-exp, when available, or relative to the median cysteine reactivity change for the corresponding protein. **c**, GO:BP analysis (Fisher’s exact test) of proteins with cysteine reactivity changes in TLR4-stimulated macrophages relative to M0 cells. Terms with FDR < 0.01 and protein number > 5 are shown. Reg., regulation. **d**, Heatmaps showing cysteine reactivity changes across macrophage stimulation conditions for proteins grouped by functional class: guanine nucleotide exchange factors (GEFs), GTPase-activating proteins (GAPs), and GTPases. Colors indicate the direction of reactivity changes relative to protein expression or median cysteine reactivity. Blank cells indicate no reactivity change. **e**, Crystal structure of CDC42 bound to a GTP analog (yellow; PDB 2QRZ^31^), with the differentially reactive cysteine C18 highlighted in red and the non-reactive cysteine C105 in grey. **f**, Box plots showing changes in CDC42 expression (triangles) and cysteine reactivity (dots) across macrophage stimulation conditions compared to M0 cells. Boxes indicate the 25th to 75th percentiles, and whiskers extend to 1.5x the interquartile range. Boxes on the bottom margin indicate cysteines classified as differentially reactive. **g**, Structure of the high-molecular-weight, cysteine-reactive maleimide probe used for Western blot - Activity-based protein profiling (WB-ABPP) experiments (top), and schematic showing potential covalent adducts formed following probe treatment, including modification of GTP-sensitive cysteines (bottom). **h**, Representative CDC42 Western blot of lysates from M0 or TLR4-stimulated macrophages treated with GTP or ATP before labeling with the maleimide probe (top), with quantification of CDC42 bands shown below. **i**, GTP abundance in M0 and TLR4-stimulated macrophages. Data in (**h**) and (**i**) represent the average ± SEM of n = 4 donors, and statistical comparison is by a two tailed t-test with p-values shown (ns, p > 0.05; *, p < 0.05).

**Fig. 3.**
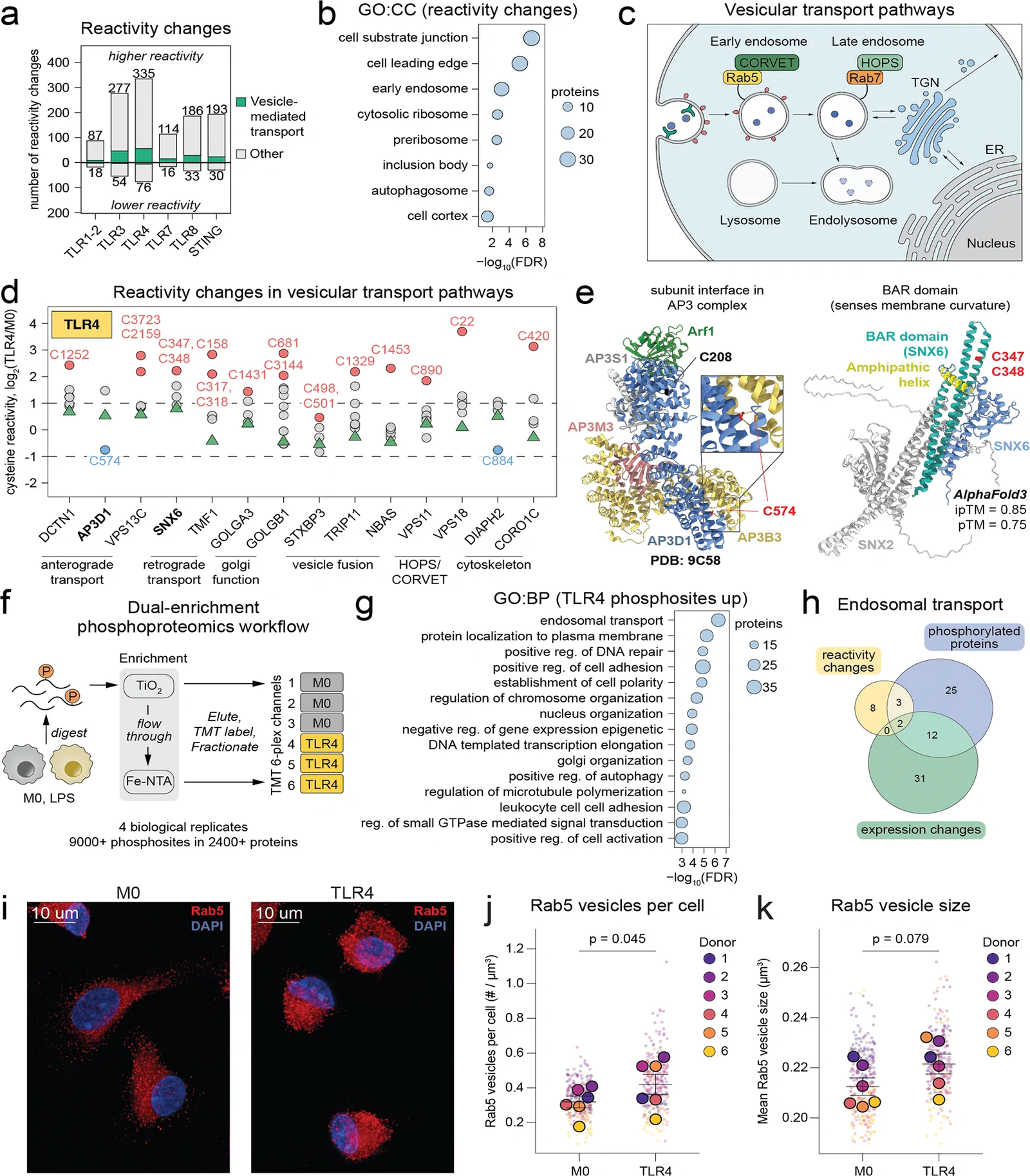
Cysteine reactivity and phosphoproteomic profiling reveal activation of vesicular trafficking pathways. **a**, Numbers of cysteines with reactivity changes across macrophage stimulation conditions relative to M0 cells (n = 3 donors). Proteins associated with vesicle-mediated transport based on Gene Ontology annotation are labeled in green. **b**, Gene ontology cellular component (GO:CC) enrichment analysis of proteins with cysteine reactivity changes in TLR4-stimulated macrophages relative to M0 cells. Terms with FDR < 0.01 and protein number > 5 are shown. Reg., regulation. **c**, Schematic showing vesicular trafficking pathways highlighting relevant organelles and select compartment-specific markers. **d**, Log_2_(fold change) in protein expression and cysteine reactivity for selected proteins in vesicular transport pathways in TLR4-stimulated macrophages relative to M0 cells. Protein expression changes are shown as green triangles and cysteine reactivity changes as dots. Cysteine reactivity and protein expression data are from n = 3 donors. **e**, Left, Cryo-EM structure of the AP3 complex containing AP3D1 (PDB 9C58^43^), with the differentially reactive cysteine C574 highlighted in red. Right, AlphaFold 3-predicted structure of SNX2-SNX6 heterodimer^44^, with C347 and C348, which are contained within the same differentially reactive tryptic peptide in SNX6, highlighted in red. **f**, Schematic of the dual-enrichment phosphoproteomics workflow that uses TiO_2_ and Fe-NTA enrichment to achieve in-depth phosphopeptide coverage. **g**, GO:BP enrichment analysis of proteins with phosphosites with significantly increased phosphorylation in TLR4-stimulated cells compared to M0 (FC > 2, p < 0.05). Terms with FDR < 0.01 and protein number > 5 are shown. **h**, Venn diagram of proteins associated with endosomal transport showing changes in cysteine reactivity (FC > 2 relative to expression value), expression (FC > 1.5, p < 0.05), or phosphorylation (FC > 2, p < 0.05) in TLR4-stimulated macrophages compared to M0 cells. **i**, Representative confocal microscopy images of Rab5 vesicles (red) with DAPI-stained nuclei (blue) in unstimulated M0 and TLR4-stimulated macrophages (Scale bar: 10 μm, zoomed: 5 μm). **j**, Quantification of number of vesicles per cell, normalized to total cell volume. Dots represent individual cells and are colored by donor. The average across cells for each donor is represented by a larger dot. Lines represent mean and SEM for n = 6 donors. Significance was determined by a two-tailed paired t-test on the donor means. **k**, Quantification of mean Rab5 vesicle size per cell. Dots represent individual cells and are colored by donor. The average across cells for each donor is represented by a larger dot. Lines represent mean and SEM for n = 6 donors. Significance was determined by a two-tailed paired t-test on the donor means.

**Fig. 4:**
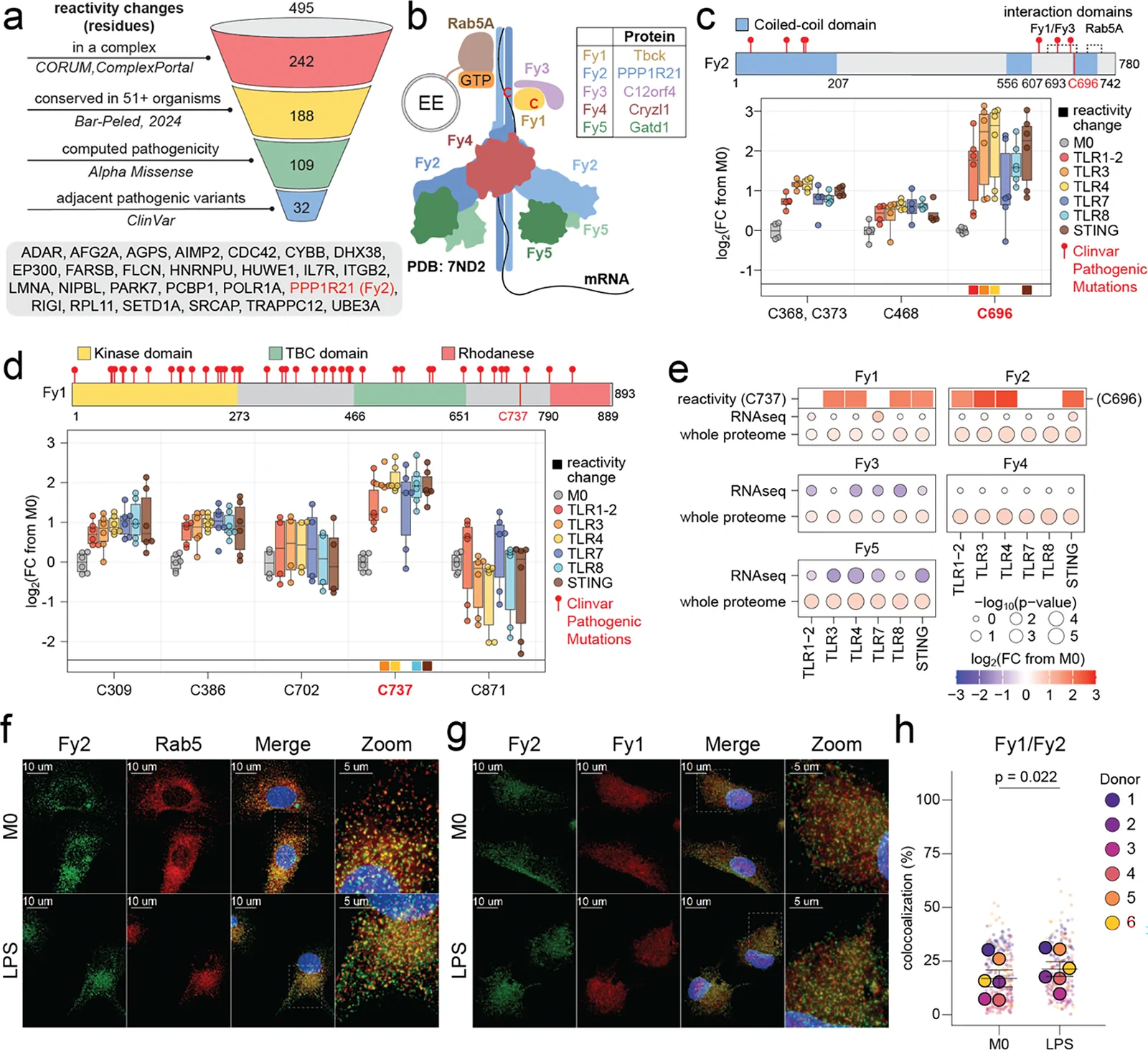
The RNA-trafficking FERRY complex assembles in macrophages and associates with early endosomes. **a**, Prioritization workflow for differentially reactive cysteines with potential functional relevance. Residues were sequentially filtered for significant reactivity changes in at least one macrophage stimulation condition (495 cysteines), localization within proteins annotated as members of protein complexes (CORUM^55, 56^ and Complex Portal^57, 58^; 242 cysteines), evolutionary conservation across 102 organisms^54^ (188 cysteines), predicted pathogenicity by AlphaMissense^53^ (109 cysteines), and proximity to pathogenic or likely pathogenic germline variants reported in ClinVar^59^ (within 15 residues; 32 cysteines). Top 32 cysteine-containing peptides mapped to 26 high-confidence protein targets, which are listed in the grey oval. **b**, Schematic of the five-membered FERRY complex^20^, with individual subunits shown in different colors. Differentially reactive cysteines in Fy1 and Fy2 are denoted as red “C” symbols. Inset lists the UniProt protein names corresponding to each FERRY complex member. **c**,**d**, Top, schematic of Fy1 and Fy2 proteins with functional domains and pathogenic ClinVar variants annotated. Bottom, box plots showing changes in Fy2 and Fy1 expression (triangles) and cysteine reactivity (dots) across macrophage stimulation conditions compared to M0 cells. Boxes indicate the 25th to 75th percentiles, and whiskers extend to 1.5x the interquartile range. Boxes on the bottom margin indicate cysteines classified as differentially reactive. **e**, Multi-omic characterization of stimulation-dependent changes in FERRY complex members. For each subunit (Fy1-Fy5), dots show the change across the profiled stimulation conditions in transcript abundance (RNA-seq) and protein abundance (TMT-exp). Cysteine reactivity changes for the indicated residues are shown as colored boxes for proteins with reactivity changes. Dot color indicates the direction and magnitude of change, and dot size represents −log_10_(p-value). **f**,**g**, Representative confocal microscopy images of Fy2 (red) and Rab5 (green; early endosome marker) (**f**) or Fy2 (red) and Fy1 (green) (**g**) in M0 and TLR4-stimulated macrophages. Gray boxes highlight zoomed-in regions. **h**, Quantification of colocalized intensity of Fy1 with Fy2. The average across cells for each donor is represented by a larger dot. Lines represent mean and SEM for n = 6 donors. Significance was determined by a two-tailed paired t-test on the donor means.

**Fig. 5.**
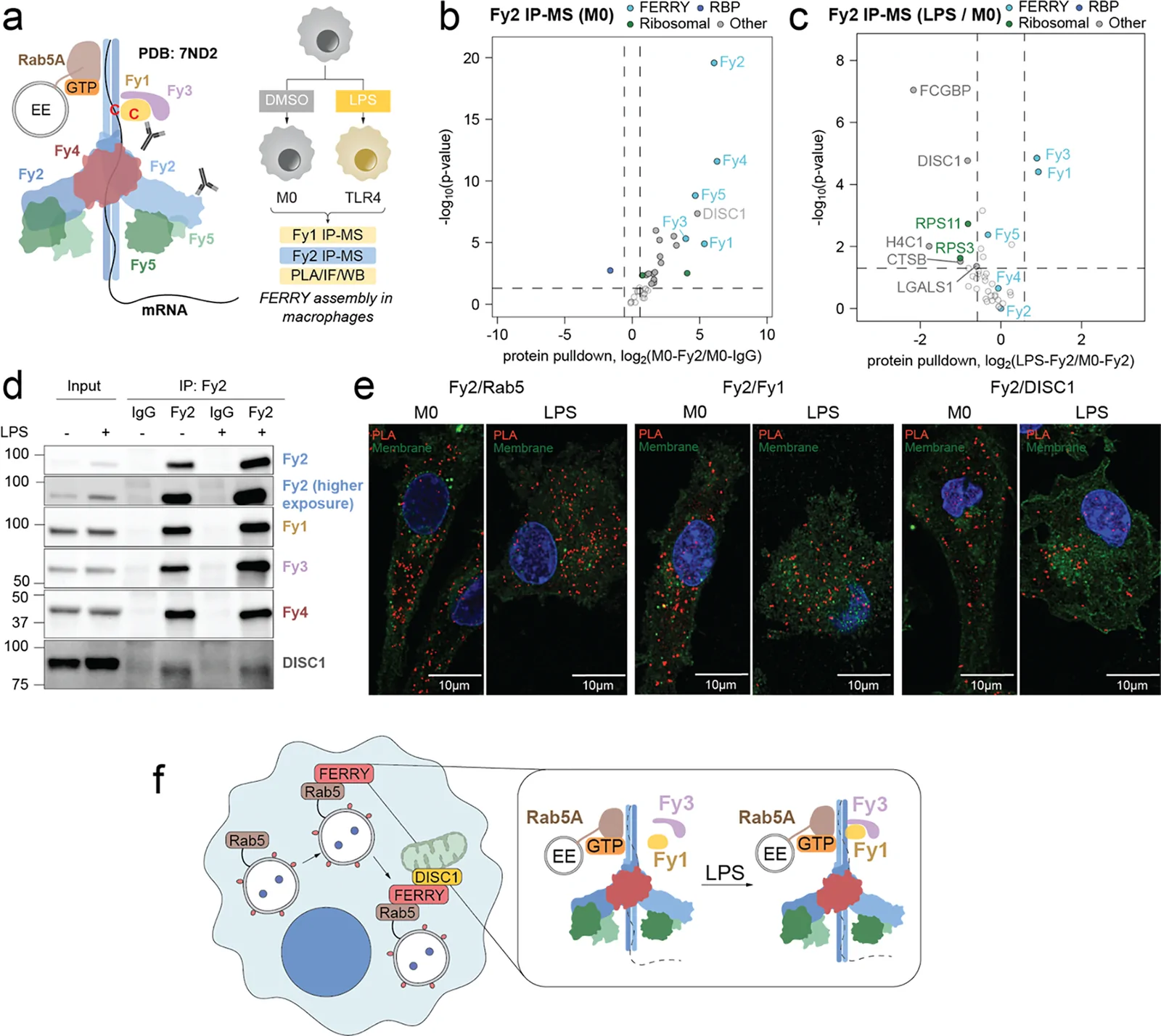
Characterization of state-dependency of FERRY complex assembly and composition. **a**, Schematic of experiments performed to investigate state-dependent differences in FERRY complex assembly and composition. Antibody symbols denote proteins targeted for IP-MS experiments. **b**, Volcano plot of CRAPome-filtered^64^ proteins co-enriched with endogenous Fy2 relative to an IgG control following immunoprecipitation from unstimulated M0 macrophages. Vertical dashed lines mark the fold-change cutoff (±log_2_(1.5)) and the horizontal dashed line marks p = 0.05. Proteins exceeding both thresholds in the upper right quadrant are considered significantly enriched. Points are colored by protein category: FERRY complex subunits (light blue); RNA-binding proteins (RBPs; Gerstberger et al., 2014^65^; dark blue); ribosomal proteins (GO annotation; green); and all other proteins (grey). **c**, Volcano plot comparing CRAPome-filtered proteins co-enriched with endogenous Fy2 following immunoprecipitation from M0 and TLR4-stimulated macrophages. **d**, Representative Western blots showing Fy2 and co-immunoprecipitated Fy1, Fy3, Fy4, and DISC1 following immunoprecipitation of endogenous Fy2 from M0 and TLR4-stimulated macrophage lysates. **e**, Representative PLA images detecting interactions between Fy2 and Rab5 (left), Fy2 and Fy1 (middle), or Fy2 and DISC1 (right) in M0 and TLR4-stimulated macrophages. PLA signals are shown in red, and cell membranes and nuclei are shown in green and blue, respectively. **f**, Proposed model of state-dependent remodeling of the FERRY complex following TLR4 stimulation in macrophages.

## Data Availability

Raw proteomic data and RNA-sequencing data will be deposited to PRIDE and NCBI GEO. The code used for data processing and visualization will be deposited to GitHub. Any additional information required to reanalyze the data reported in this paper is available from the lead contact upon request.
