# Supplementary material for: Chemical proteomics reveals post-translational remodeling of endosomal pathways during macrophage activation
Source: bioRxiv. 2026 Oct 1:2026.09.25.754469. Preprint. [Version 1] doi: 10.64898/2026.09.25.754469 (PMC13648533; doi:10.64898/2026.09.25.754469)
Supplement: Supplementary file 1 [file NIHPP2026.09.25.754469v1-supplement-1.pdf]

bioRxiv preprint doi: https://doi.org/10.64898/2026.09.25.754469; this version posted October 1, 2026. The copyright holder for this preprint (which was not certified by peer review) is the author/funder, who has granted bioRxiv a license to display the preprint in perpetuity. It is made available under a CC-BY 4.0 International license.

Supplementary Information.

(A) Supplementary Data Set Legends

Data S1

#	Title	Description
1	S1-1 Bulk RNA Sequencing Data	Differential gene expression analysis of bulk RNA-seq data from M0 and TLR/STING-stimulated macrophages (related to Figure 1 and Extended Data Fig. 1, 2).
2	S1-2 qPCR Primer Sequences	Sequences of primers for qPCR for validation of RNA-seq (related to Extended Data Fig. 2)

Table S1-1: Differential gene expression analysis was performed with DESeq2, using its median-of-ratios method to normalize RNA counts. Genes with zero mapped reads were excluded from display. Each stimulus was compared against M0 (denoted M0_Low in the corresponding GEO submission). Normalized counts for each stimulus and donor are shown to the right of the differential expression statistics. Genes are the union across the seven comparisons. Genes are restricted to the primary assembly. Data are from n = 3 donors.

Table S1-2: Forward and reverse primers for validation of RNA-seq targets and housekeeping genes by qPCR.

Data S2

#	Title	Description
1	S2-1 Unenriched proteomics	Whole proteome data from M0 and TLR/STING-stimulated macrophages (related to Figure 1 and Extended Data Fig. 1, 2)
2	S2-2 Bulk RNA vs WP	Whole proteome and transcriptome data from M0 and TLR/STING-stimulated macrophages (related to Figure 1 and Extended Data Fig. 1, 2)
3	S2-3 GSEA of PC loadings	Gene set enrichment analysis (GSEA) of whole proteome PCA loadings (related to Extended Data Fig. 1)
4	S2-4 GO-term enrichment	GO-term enrichment analysis of proteomic data (related to Figures 2 and 3, and Extended Data Fig. 2, 5, and 7)
5	S2-5 Reactivity changes	Cysteine reactivity changes identified from IA-DTB reactivity TMT-ABPP data (related to Figures 2, 3, and 4, and Extended Data Fig. 3, 4, 5, and 6).
6	S2-6 Phosphoproteomics	Expression-normalized phosphoproteomics data from M0 and LPS-stimulated macrophages (related to Figure 3 and Extended Data Fig. 5)

bioRxiv preprint doi: https://doi.org/10.64898/2026.09.25.754469; this version posted October 1, 2026. The copyright holder for this preprint (which was not certified by peer review) is the author/funder, who has granted bioRxiv a license to display the preprint in perpetuity. It is made available under a CC-BY 4.0 International license.

7	S2-7 Immunoprecipitation MS	Immunoprecipitation-mass spectrometry (IP-MS) data (related to Figure 5 and Extended Data Fig. 7)
8	S2-8 Cross-omics Venn	Per-protein membership behind the cross-omics GO-term Venn diagrams (related to Figure 3 and Extended Data Fig. 5)

Table S2-1: TMT-exp data showing protein expression changes in macrophages stimulated with TLR/STING agonists compared to M0 macrophages. Data are from n = 3 donors.

Table S2-2: TMT-exp and bulk RNA-seq data showing gene and protein expression changes in macrophages stimulated with TLR/STING agonists compared to M0 macrophages. TMT-exp and bulk RNA-seq are from n = 3 donors each (different donors for each assay).

Table S2-3: Pre-ranked GSEA of the protein loadings on each principal component of the whole proteome PCA, against the GO biological process gene sets of the 2026 MSigDB. A positive NES marks a term enriched at the positive end of that principal component. Only significant terms (FDR q-value < 0.01) are shown.

Table S2-4: GO-term enrichment analysis of whole proteome, reactivity, and phosphoproteomic data. Fisher's exact test and the 2026 MSigDB gene sets were used for all analyses, and the background for each test is specified in the table. Only terms reaching significance (FDR < 0.05 and more than 5 proteins, except where a panel's own cutoff is stricter) are listed, so a panel may plot a non-significant point that has no row here. 'Relevant figure' gives the panel in which each term is plotted. Protein counts for the Cross-omics rows are the same change sets the Venn diagrams use; see Table S2-8 for their per-protein membership.

Table S2-5: Proteins with identified cysteine reactivity changes in at least one condition (TLR1-2, TLR3, TLR4, TLR7, TLR8, STING) compared to M0 macrophages. Data are median from n = 3 donors. Per-condition columns list one value per residue in the 'residue' column, '|'-separated; '(C1;C2)' marks a change that could not be attributed to a single residue and counts once. The cross-reference columns on the right describe only the cysteines that change: complex membership (CORUM, ComplexPortal), conservation depth (out of 102 organisms), AlphaFold solvent accessibility (pPSE; buried > 9, intermediate > 5) and secondary structure, AlphaMissense pathogenicity (pathogenic > 0.564, benign < 0.34), and pathogenic ClinVar variants within 15 residues.

Table S2-6: Phosphosite quantification in macrophages stimulated with LPS (TLR4) compared to M0 macrophages. Per-replicate channel ratios were normalized to protein expression by dividing each phosphosite's channel ratio by that protein's whole proteome fold change, so changes reflect phosphorylation rather than protein abundance; phosphosites on proteins without whole proteome coverage are not reported. Data are from n = 4 donors x 3 technical replicates, restricted to phosphosites quantified in at least 2 donors. Significance is an unpaired two-sample t-test of all TLR4 channels against all M0 channels; p-values are raw and are not corrected for multiple testing. 'Regulation' marks a phosphosite Significant Up or Significant Down at p < 0.05 and a fold change greater than 2.

Table S2-7: Immunoprecipitation-mass spectrometry showing proteins co-enriched with TBCK or PPP1R21 in LPS-stimulated macrophages (TLR4) or unstimulated (M0) macrophages. Data are from n = 3 donors (different donors for each immunoprecipitation assay).

bioRxiv preprint doi: https://doi.org/10.64898/2026.09.25.754469; this version posted October 1, 2026. The copyright holder for this preprint (which was not certified by peer review) is the author/funder, who has granted bioRxiv a license to display the preprint in perpetuity. It is made available under a CC-BY 4.0 International license.

Table S2-8: One row per protein in the union of the three TLR4-vs-M0 change sets for each GO term, with a 1/0 flag per profiling method. Unlike Table S2-4, these are raw overlaps of the term's gene list with each method's significant changes, not an enrichment test, so no p-values apply; summing a flag column gives that method's circle total in the panel. 'GO term source' gives the gene set used, which for GTPase activity is a union of GTPase GO terms rather than GO:0003924 alone.

Data S3

#	Title	Description
1	S3-1 Polar metabolites	Abundance of polar metabolites in M0 and TLR4-stimulated macrophages (related to Figure 2 and Extended Data Fig. 3)

Table S3-1: Polar metabolite abundance values in TLR4-stimulated macrophages compared to M0 macrophages as determined by LC-MS/MS analysis. The channel ratio values after quantile regression imputation of left censored data (QRILC) are shown alongside raw signal intensity values and differential abundance calculations. Data are from n = 4 donors.

Data S4

#	Title	Description
1	S4-1 IF per-cell measurements	Per-cell immunofluorescence measurements of Fy2 with Fy1, Fy4, and Rab5 in M0 and TLR4-stimulated macrophages (related to Figure 3, 4, and Extended Data Fig. 6)
2	S4-2 IF colocalization stats	Donor-level colocalization of Fy2 with Fy1, Fy4, and Rab5 in M0 and TLR4-stimulated macrophages (related to Figure 4 and Extended Data Fig. 6)

Table S4-1: Per-cell vesicle counts, sizes, and intensities, and split colocalization coefficients of Fy2 with Fy1, Fy4, or Rab5 in M0 and TLR4-stimulated macrophages, one row per cell. In column headers, the partner of Fy2 in each antibody pair is referred to as "marker". Colocalization coefficients are shown in both directions: Coloc. (X/Y) is the fraction of the X signal on Y. Puncta with a radius < 0.15 µm were excluded. No chromatic aberration correction was applied. Data are from n = 6 donors.

Table S4-2: Donor-level split colocalization coefficients for each direction of each antibody pair (intensity-weighted). Per-cell values were averaged per donor and condition, and the mean ± SEM of donor means is shown; P values are from a two-tailed paired t-test on the donor means. The number of cells quantified per condition is listed for each pair. Data are from n = 6 donors.

bioRxiv preprint doi: https://doi.org/10.64898/2026.09.25.754469; this version posted October 1, 2026. The copyright holder for this preprint (which was not certified by peer review) is the author/funder, who has granted bioRxiv a license to display the preprint in perpetuity. It is made available under a CC-BY 4.0 International license.

(B) Biological Methods

EXPERIMENTAL MODEL AND SUBJECT DETAILS

Human samples

All studies involving primary human macrophages were performed using buffy coats obtained from the New York Blood Center, leukopaks and LRS cones from STEMCELL Technologies, and blood and leukapheresis samples from the Rockefeller University Hospital. Blood samples were collected from randomly selected, de-identified healthy donors with donor sex not recorded. The human subjects protocol was reviewed and approved annually by the Rockefeller University Hospital Institutional Review Board (IRB; protocol EVI-1024).

Isolation of human peripheral blood mononuclear cells (PBMCs) and monocytes

Peripheral blood was diluted with PBS, and 25 mL of the diluted cells were layered over 12.5 mL of Lymphoprep (STEMCELL Technologies) using 50 mL Falcon tubes. Tubes were centrifuged at room temperature (900 x *g*, 25 °C, 20 min) with the acceleration set to 50% and brake disabled. Following the spin, the plasma and cell layers were transferred to new 50 mL Falcon tubes, diluted with PBS, and pelleted (520 x *g*, 25 °C, 8 min). The pellet was washed once in PBS, resuspended in 5 mL of 1X RBC lysis buffer (Santa Cruz Biotechnologies), and incubated for 5 min at room temperature. Cells were then diluted with PBS, pelleted (520 x *g*, 25 °C, 8 min), and washed with PBS. PBMCs were resuspended at 50×10^6 cells /mL, and monocytes were isolated by negative selection using the EasySep Monocyte Enrichment Kit without CD16 Depletion (STEMCELL Technologies) according to manufacturer's instructions.

METHODS DETAILS

Macrophage isolation and polarization

Freshly isolated monocytes were resuspended at 1×10^6 cells/mL in macrophage media, consisting of RPMI-1640 supplemented with 10% FBS (Omega Scientific), 2 mM GlutaMAX (Gibco), 100 U/mL Penicillin-Streptomycin (Gibco), 1 mM Sodium Pyruvate (Gibco), 2 mM HEPES pH 7.0 (Boston Bioproducts), and 50 ng/mL mCSF (STEMCELL Technologies). Cells were seeded at 15×10^6 cells per 15 cm culture plate, 5×10^6 cells per 10 cm culture plate, or 0.83×10^6 cells per well of a 6-well plate and cultured at 37 °C in a humidified 5% CO_2 incubator for three days. Cells were washed three times with PBS and re-supplemented with macrophage media containing mCSF for another three days. Cells were then washed three times with PBS and re-supplemented with macrophage media containing mCSF and TLR or STING agonists (see table below) for 24 h. Cells were then placed on ice, washed 3x with ice-cold PBS, and harvested into ice-cold PBS by scraping. Cells were then pelleted (520 x *g*, 4 °C, 8 min), flash frozen, and kept at -80 °C until further analysis.

Target	Agonist	Concentration	Company	Catalog #
TLR1-2	CU-T12-09	10 µM	Millipore Sigma	5325830001
TLR3	Poly(I:C)	20 µg/mL	Tocris Bioscience	42-871-0
TLR4	LPS	100 ng/mL	Millipore Sigma	L2630-10
TLR7	Imiquimod	10 µg/mL	Fisher Scientific	AAJ63990MC
TLR8	CL075	0.5 µg/mL	Millipore Sigma	SML2235

bioRxiv preprint doi: https://doi.org/10.64898/2026.09.25.754469; this version posted October 1, 2026. The copyright holder for this preprint (which was not certified by peer review) is the author/funder, who has granted bioRxiv a license to display the preprint in perpetuity. It is made available under a CC-BY 4.0 International license.

TLR9	CpG ODN 2006	5 µg/mL	Integrated DNA Technologies	custom
STING	cGAMP	10 µg/mL	InvivoGen	tlrl-nacga23-5

Flow cytometry analysis

Polarized cells were washed once with room temperature PBS and incubated with TrypLE (Gibco, 12604021) for 30 min at 37 °C in a humidified 5% CO_2 incubator, with vigorous tapping on the edges of the plate every 10 min. Cells were then gently scraped and transferred into a tube. The plate was then washed with PBS, and the wash was pooled with previously collected cells. Cells were spun down (520 x *g*, 4 °C, 8 min), resuspended in PBS, and plated at 100,000 live cells per well in a U-bottom 96-well plate. Cells were spun down (520 x *g*, 4 °C, 5 min) and washed once with PBS. Cells were then resuspended in Live/Dead Fixable NIR Viability Dye (Invitrogen, L10119) at a 1:50,000 dilution in PBS, and incubated at room temperature for 15 min in the dark. Cells were then spun down and washed once with PBS. Cells were then incubated with 25 µL of Fc Block (BioLegend, 422302) and Monocyte Block (BioLegend, 426102) both at a 1:20 dilution in staining buffer (1% FBS in PBS) for 15 min at 4 °C. Cells were then stained for surface markers at a 1:400 dilution in Brilliant Stain buffer and incubated on ice for 25 min at 4 °C. Cells were then diluted with 150 µL of staining buffer, spun down, and washed once more with staining buffer. Cells were then resuspended in 4% PFA (FUJIFILM) for 15 min at 4 °C. Cells were spun down, washed once with staining buffer, and resuspended in staining buffer with 2 mM EDTA. Data was acquired using an Agilent Novocyte Penteon cytometer, with a minimum of 4,000 live cells measured per condition. Data was reported as geometric mean of individual markers with a gate set using fluorescence minus one (FMO) controls and normalized to the M0 condition. Significance was calculated by a matched one-way ANOVA with Dunnett's multiple comparisons test (GraphPad Prism, Version 10).

Staining type	Marker	Fluorophore	Catalog #	Clone	Dilution
Surface	CD14	BV650	563419	M5E2	1:400
Surface	CD11b	BV711	563168	M1/70	1:400
Surface	CD80	PE	560925	L307.4	1:400
Viability	Viability	Live/Dead NIR	L10119	--	1:50,000

Enzyme-Linked Immunosorbent Assay (ELISA)

Following the 24 h incubation with TLR or STING agonists, macrophage media was taken from cells into a new 96-well plate, spun briefly (520 x g, 4 °C, 5 min) to remove cell debris, and the supernatant containing cytokines was stored at -80 °C until analysis. ELISA was performed with IL-6 or TNFα DuoSet ELISA Kits (R&D Systems, DY206 and DY210) according to manufacturer's instructions. Absorbance was read out on a plate reader at both 450 nm with a 540 nm background subtraction, and concentrations were interpolated from standards fit to a 4-PL model. Significance was calculated by a matched one-way ANOVA with Dunnett's multiple comparisons test (GraphPad Prism, Version 10) on log_2-transformed concentration values.

bioRxiv preprint doi: https://doi.org/10.64898/2026.09.25.754469; this version posted October 1, 2026. The copyright holder for this preprint (which was not certified by peer review) is the author/funder, who has granted bioRxiv a license to display the preprint in perpetuity. It is made available under a CC-BY 4.0 International license.

RNA isolation for qPCR and RNA sequencing analysis of basal state and activated macrophages

Following stimulation, plates containing cells were put on ice, washed three times with ice-cold PBS, and the cells were harvested by scraping into ice-cold PBS and transferring to new Falcon tubes. Cells were then pelleted (520 x g, 4 °C, 5 min), transferred to new low binding Eppendorf tubes, resuspended in TRIzol reagent (Invitrogen, 15-596-026), snap-frozen in liquid nitrogen, and stored at -80 °C. RNA was extracted using the Direct-zol RNA Miniprep kit (Zymo Research, R2052) with on-column DNase I digestion according to manufacturer's instructions. Purified RNA was flash frozen and stored at -80 °C until further analysis.

RNA sequencing

Sample preparation

RNA quality and concentration were assessed using an Agilent Bioanalyzer, and samples with a RIN > 8 were used for the library prep. 100 ng of total RNA was used to generate RNA-seq libraries with the Illumina stranded mRNA kit (20040534) according to the manufacturer's protocol. Libraries were prepared with unique dual indexes and pooled at equal molar ratios. The pool was denatured and sequenced on an Illumina NovaSeq 6000 sequencer using V1.5 reagents, S2 flowcell, and NovaSeq Control Software V1.7.0 to generate 2 x 100 bp paired-end reads, according to the manufacturer's protocol.

Data processing

Paired-end RNA-seq reads were aligned to the GRCh38 reference genome using STAR (v2.7.7a) in two-pass mode. The STAR genome index was generated using GENCODE release 44 (Ensembl 110) gene annotations. Gene-level counts were generated with featureCounts (v1.4.6-p5) in paired-end mode using the corresponding GENCODE release 44 annotations.

Principal Component Analysis

The 5,000 protein-coding genes with the highest row-wise variance were used and normalized count values were $\log_2$-transformed before principal component analysis (PCA). PCA was performed using *scikit-learn* library for Python. The first two principal components (PC1 and PC2) were plotted, and loadings were visualized for the five genes with the highest and lowest loadings along each component.

RT-qPCR

RNA concentration was quantified using a Qubit fluorometer with the Qubit RNA Broad Range (BR) kit (Invitrogen, Q10210). cDNA was synthesized using the iScript cDNA Synthesis Kit (Bio-Rad, 1708891) according to manufacturer's instructions. qPCR was performed on a QuantStudio5 (Thermo Fisher) in standard cycling mode with a terminal melt curve step in a 384-well plate. Each reaction contained 50 ng of cDNA input, 400 nM of each primer, and 1X Select Master Mix (Applied Biosystems, 4472908) at a final volume of 10 µL per well. Primer sequences are listed in Supplementary Table S1-2. Samples were run in triplicate and quantified by the standard curve method. No-template (NTC) and no-reverse transcriptase (-RT) controls were included on each plate. Gene expression was normalized to the average of housekeeping genes ACTB and C1orf43, which were chosen due to their unchanging expression in our RNA sequencing data, and expressed as $\log_2$(fold change) relative to unstimulated (M0) control conditions. Statistical comparisons were

bioRxiv preprint doi: https://doi.org/10.64898/2026.09.25.754469; this version posted October 1, 2026. The copyright holder for this preprint (which was not certified by peer review) is the author/funder, who has granted bioRxiv a license to display the preprint in perpetuity. It is made available under a CC-BY 4.0 International license.

performed by one-way ANOVA with post-hoc multiple comparisons testing (GraphPad Prism, Version 10)

Metabolomic analysis

Sample preparation

Monocytes were plated on 10 cm plates and differentiated as described above. Macrophages were then polarized with either fresh macrophage media or media supplemented with agonists for 24 h. Plates were then transferred to ice and washed twice with ice-cold PBS. Cells were scraped directly into 1 mL of ice-cold 80% MeOH in water and transferred to an Eppendorf tube. Cells were then vortexed for 45 s and incubated on dry ice for 5 min. Cells were again vortexed for 45 s and pelleted to get rid of debris (14,000 x *g*, 4 °C, 20 min). Supernatant was transferred to a clean tube and dried under flow of Nitrogen gas. Samples were stored at -80 °C until analysis.

Polar metabolite analysis

The sample was redissolved in HPLC grade water before it was analyzed by LC-MS/MS. Metabolites were measured on an Orbitrap Exploris 240 mass spectrometer (Thermo Scientific) coupled to a Vanquish UPLC system (Thermo Scientific). A Sequant ZIC-pHILIC column (2.1 mm i.d. × 150 mm, particle size of 5 µm, Millipore Sigma) was used for separation of metabolites. A 2.1 × 20 mm guard column with the same packing material was used for protection of the analytical column. Flow rate was set at 150 µL/min. Buffers consisted of 100% acetonitrile for mobile phase A, and 0.1% NH_4OH/20 mM CH_3COONH_4 in water for mobile phase B. The chromatographic gradient ran from 85% to 30% A in 20 min followed by a wash with 30% A and re-equilibration at 85% A. The Orbitrap Exploris 240 was operated in full scan, polarity-switching mode with the following parameters: the spray voltage is 3.0 kV in positive mode and 2.5 kV in negative mode, the heated capillary temperature 275 °C, the HESI probe temperature 320 °C, the sheath gas flow 35 units, the auxiliary gas flow 7 units. MS data acquisition was performed in the m/z range of 70–1,050, with 90,000 resolution (at 200 m/z). The AGC target was 1e6 and the maximum injection time was 250 ms. The MS data was processed using XCalibur 4.1 (Thermo Scientific) to obtain the metabolite signal intensities. Identification required exact mass (within 5 ppm) and standard retention times.

Data processing and imputation

Within each biological replicate, metabolites were required to have at least one condition with an average signal intensity above 5,000 (179/205 metabolites passed). After filtering and log transformation, missing data was imputed through quantile regression imputation of left-censored data (QRILC) performed using imputeLCMD version 2.1. Channel ratios (signal intensity / sum of signal intensities per metabolite) were calculated and used for all analyses.

Hierarchical clustering

The top 25 increasing and decreasing metabolites by p-value (T-test between M0/TLR4) were used for hierarchical clustering. A z-score was calculated for each observation by $z = (x-\mu)/\sigma$, where x is the channel ratio, μ is the mean channel ratio of the metabolite across all replicates, and σ is the standard deviation of the channel ratio of the metabolite across all replicates. Hierarchical clustering was performed using ComplexHeatmap[1] library version 2.10.0 for R version 4.1.1. The distance matrix was calculated using the "euclidean" method and clustering was performed using the "complete" method. Metabolite class annotations were curated through literature review.

bioRxiv preprint doi: https://doi.org/10.64898/2026.09.25.754469; this version posted October 1, 2026. The copyright holder for this preprint (which was not certified by peer review) is the author/funder, who has granted bioRxiv a license to display the preprint in perpetuity. It is made available under a CC-BY 4.0 International license.

Proteomic platforms: Abundance-based proteomics with TMT multiplexing

Sample preparation

Three 16-plex experiments were performed with primary human macrophages from different donors, consisting of technical duplicates from 8 different macrophage states. Frozen cell pellets were thawed on ice and resuspended in PBS with protease inhibitor cocktail (Roche cOmplete Mini, EDTA-free, 1 tablet per 10 mL PBS). Cells were then lysed with sonication (three cycles of 8 x 1s pulses on a 60% duty cycle). Protein concentration was determined with the DC assay (Bio-Rad), and lysates were normalized to 2 mg/mL.

For each sample, 100 µL (200 µg) of lysate was added to 48 mg urea to a final concentration of 8 M. Freshly made stock of DTT (5 µL, 200 mM in water) was added to each sample and incubated at 65 °C for 15 min. Freshly made stock of iodoacetamide (5 µL, 400 mM in water) was then added and incubated for 30 min at 37 °C in the dark with shaking. Proteins were precipitated by addition of 500 µL ice-cold methanol, 400 µL water, and 150 µL chloroform, followed by centrifugation (10,000 x *g*, 4 °C, 10 min). The upper aqueous layer was carefully removed without disturbing the protein disc, and an additional 400 µL of cold methanol was added. Pellets were resuspended by sonication and centrifuged (10,000 x *g*, 4 °C, 10 min). The supernatant was discarded, and the pellets were resuspended by sonication in 200 mM EPPS buffer (pH 8.0). The proteins were then digested with 2.5 µg of Trypsin/LysC (Promega) with $CaCl_2$ (1 mM final concentration) overnight at 37 °C with shaking.

TMT labeling

Digests were centrifuged at room temperature (10,000 x *g*, 25 °C, 5 min) to remove undigested material and supernatants were transferred to new tubes. Peptide concentration was measured with the microBCA assay (Thermo Fisher, 23235). For each sample, 25 µg of peptides were then transferred to a new tube, topped up to equal volumes, and acetonitrile was added to each tube to a final concentration of 25%. Samples were then labeled with 100 µg of corresponding TMT tags (Thermo Fisher, A44521; 20 µg/µL in acetonitrile) for 1 h at room temperature. Labeling reactions were quenched with 6 µL of 5% Hydroxylamine in water (Sigma-Aldrich, 467804) for 15 min at room temperature and acidified with formic acid (VWR, 94318) to a final concentration of 5%.

Normalization of TMT input and desalting

A ratio check sample was prepared by combining a small aliquot from each labeled sample, desalting using a C18 stage tip (Thermo Fisher), and drying it in a SpeedVac. The sample was resuspended in Buffer A (5% acetonitrile, 95% water, 0.1% formic acid) and analyzed using an Orbitrap Eclipse mass spectrometer using a 70-min LC gradient: 0-10 min, 5% buffer B (0.1% FA in acetonitrile) in buffer A (0.1% FA in water); 10-50 min, linear increase from 5% to 20% buffer B; 50-55 min, ramp to 45% buffer B; 55-57 min, ramp to 95% buffer B; 57-59 min, hold at 95% buffer B; 59-61 min, decrease to 5% buffer B; 61-63 min, ramp back to 95% buffer B; 63-70 min: re-equilibration at 5% buffer B. Normalization factors (N) were calculated for each TMT channel, and volume equivalents of 10 µL x N per channel were pooled and dried in the SpeedVac. The pooled sample was then resuspended in Buffer A and acidified with formic acid to a final pH of < 3. A C18 SepPak cartridge (Waters, WAT054955) was activated with 3 x 1 mL of acetonitrile and equilibrated with 3 x 1 mL of Buffer A. Acidified sample was added to the equilibrated column, and the flow-through was reloaded for maximum enrichment. The column was then washed three

bioRxiv preprint doi: https://doi.org/10.64898/2026.09.25.754469; this version posted October 1, 2026. The copyright holder for this preprint (which was not certified by peer review) is the author/funder, who has granted bioRxiv a license to display the preprint in perpetuity. It is made available under a CC-BY 4.0 International license.

times with Buffer A, eluted in 1 mL of Buffer B (80% acetonitrile, 20% water, 0.1% formic acid), and dried on the SpeedVac.

High pH HPLC fractionation (general protocol for all LC-MS/MS/MS workflows)

High pH HPLC fractionation was performed following previously published protocols[2]. Briefly, the desalted peptides were resuspended in 500 µL of Buffer A and fractionated offline using an Agilent HPLC system into a 96-well plate containing 10 µL of 20% formic acid. Separation was performed on a Zorbax Extend-C18 column (3.5 µm, 4.6 × 250 mm) at a flow rate of 0.5 mL/min using a 10 mM ammonium bicarbonate (Buffer C) to acetonitrile (Buffer D) gradient over 125 min. The gradient is as follows: 0-2 min, 100% Buffer C; 2-3 min, 0% to 13% Buffer D; 3-60 min, 13% to 50% Buffer D; 60-70 min, 50% to 80% Buffer D; 71-75 min, 100% Buffer D; 75-76 min, 100% to 0% Buffer D; 76-85 min, 100% Buffer C; 85-88 min, 0% to 13% Buffer D; 88-90 min, 13% to 80% Buffer D; 90-95 min, hold at 80% Buffer D; 96-101 min, 100% Buffer C; 101-104 min, 0% to 13% Buffer D; 104-106 min, 13% to 80% Buffer D; 106-111 min, hold at 80% Buffer D; 111-112 min, 80% to 0% Buffer D. After fractionation, the plate was dried in a SpeedVac. The peptides in each well were then resuspended in Buffer B, and the fractions were consolidated into 12 final fractions by combining wells in each column of the plate. Fractions were dried in the SpeedVac and used for LC-MS/MS/MS analysis.

General protocol for liquid chromatography mass spectrometry analysis of TMT-multiplexed samples

TMT-labeled peptides were analyzed by LC-MS/MS/MS using a 160-min gradient on an Orbitrap Eclipse mass spectrometer (Thermo Fisher) as described before[2, 3]. MS1 spectra were acquired in the Orbitrap at a resolution of 120,000 over a mass range of 400-1600 m/z, with a 40% RF lens setting, automatic gain control (AGC) target set to 250% (normalized), and maximum injection time set to automatic. Dynamic exclusion was enabled with a repeat count of 1 and an exclusion duration of 60 s. The ten most abundant precursor ions from the MS1 scan were selected for MS2/MS3 analysis, isolated using the quadrupole with a 0.7 m/z isolation window, and fragmented by collision-induced dissociation (CID) in the ion trap (standard AGC, 35% normalized collision energy (NCE), and a 120 ms maximum injection time). Synchronous precursor selection (SPS) was enabled to select up to 10 MS2 fragment ions for MS3 analysis. MS3 spectra were acquired in the Orbitrap following high-energy collision-induced dissociation (HCD) with 55% NCE, a normalized AGC target of 500%, a maximum injection time of 118 ms, a resolution of 60,000, and an 0.7 m/z isolation window for MS3.

Raw data files were processed using RAW Converter (v1.1.0.22; available at github.com/proteomicsyates/RawConverter), and the resulting MS1, MS2, and MS3 files were uploaded to the Integrated Proteomics Pipeline (IP2). Peptide identification was performed using the ProLuCID algorithm against a reverse concatenated, non-redundant version of the Human UniProt database (release 2024_03). Cysteine residues were searched with a static modification for carbamidomethylation (+57.02146 Da). A differential modification for iodoacetamide desthiobiotin labeling (+398.25292 Da) was included for site-of-labeling experiments. TMT labeling was considered as a static modification on peptide *N*-termini and lysine residues (+229.1629 Da for TMT 10-plex, +304.2071 Da for TMT 16-plex). Search results were filtered using DTASelect (v2.0) to maintain a peptide-level false discovery rate (FDR) below 1%. Quantification was performed using MS3 reporter ions with a mass tolerance of 20 ppm via the IP2 platform.

bioRxiv preprint doi: https://doi.org/10.64898/2026.09.25.754469; this version posted October 1, 2026. The copyright holder for this preprint (which was not certified by peer review) is the author/funder, who has granted bioRxiv a license to display the preprint in perpetuity. It is made available under a CC-BY 4.0 International license.

Data processing and analysis for TMT-exp experiments

Data processing

Three 16-plex experiments with primary human macrophages from different biological donors were processed. Proteins were required to have at least two unique quantified peptides to pass into the final list. Peptide ratios (signal intensity/sum of signal intensities per peptide) were calculated and ratios of all peptides per protein were averaged. Keratins and contaminants were not included in analysis. Proteins needed to be quantified in at least 2 experiments to be included in the final table.

Principal Component Analysis

Principal component analysis was performed in Python using the scikit-learn library[4]. Protein ratio values were log_2-transformed, and the analysis was restricted to proteins quantified across all experiments. The first two principal components (PC1 and PC2) were plotted, and loadings were visualized for the five proteins with the highest and lowest loadings along each component.

Gene set enrichment analysis (GSEA) of PCA loadings

PCA loadings were used as the ranking metric for GSEA, performed with the pre-rank function in GSEApy v1.1.9 against the Gene Ontology biological process database ("c5.go.bp"). The top 15 enriched terms, ranked by normalized enrichment score (NES), were plotted.

Protein half-life analysis

Protein half-lives were referenced from the monocyte turnover measurements in Kosiński *et al*[5]. Each protein's half-life was defined as the mean of two biological replicates (or the single available value), and mapped to the macrophage differential-expression values by gene symbol. Half-lives were binned (0-24, 24-48, 48-96, 96+ h) and their distribution was analyzed based on the observed concordance between transcript- and protein-abundance changes for both upregulated and downregulated proteins separately.

Hierarchical clustering

Whole-proteome TMT signals were calculated and used as log_2 fold changes relative to the mean of the M0 channels, and gene-set proteins quantified across all replicates were displayed with ComplexHeatmap. Rows were ordered by hierarchical clustering (Euclidean distance, complete linkage); columns were grouped by agonist condition.

Proteomic platforms: Cysteine reactivity profiling, TMT-ABPP

Sample preparation

Three 16-plex experiments were performed with primary human macrophages from different donors, consisting of technical duplicates from 8 different macrophage states. Frozen cell pellets were thawed on ice and resuspended in PBS. Cells were then lysed with sonication (three cycles of 8 x 1 s pulses on a 60% duty cycle). Protein concentration was determined with the DC assay, and lysates were normalized to 2 mg/mL.

For each sample, 500 µL (1 mg) of lysate was transferred into low-bind Eppendorf tubes and labeled with 5 µL of 10 mM iodoacetamide-desthiobiotin probe (IA-DTB) for 1 h at room temperature. Proteins were then precipitated by adding 500 µL ice-cold methanol and 100 µL chloroform to each tube, vortexing, and centrifuging (10,000 x *g*, 10 min, 4 °C). The supernatant

bioRxiv preprint doi: https://doi.org/10.64898/2026.09.25.754469; this version posted October 1, 2026. The copyright holder for this preprint (which was not certified by peer review) is the author/funder, who has granted bioRxiv a license to display the preprint in perpetuity. It is made available under a CC-BY 4.0 International license.

was removed without disturbing the protein disc, and an additional 400 µL of cold methanol was then added to each sample. The pellets were resuspended by sonication, centrifuged (10,000 x *g*, 10 min, 4 °C), and resuspended by sonication in 90 µL of 50 mM TEAB buffer (pH 8.5) containing 8 M urea and 10 mM DTT. Samples were then heated at 65 °C for 20 min, followed by addition of freshly prepared 10 µL of 500 mM iodoacetamide in water and incubation for 30 min at 37 °C in the dark with shaking.

Trypsin/LysC digestion and streptavidin enrichment

Samples were diluted with 300 µL of 50 mM TEAB, and proteins were digested overnight at 37 °C with shaking using 2 µg of Trypsin/LysC and 4 µL of 100 mM $CaCl_2$. The next day, Streptavidin Agarose Beads (50 µL per sample, Pierce, 20353) were washed twice with Wash Buffer 1 (WB1: 50 mM TEAB, 150 mM NaCl, 0.2% NP-40), resuspended in 300 µL WB1 per sample, and added to the digested samples. The samples were rotated at room temperature for 3 h. After enrichment, the samples were transferred to BioSpin columns (Bio-rad), washed three times each with Wash Buffer 2 (WB2: 50 mM TEAB, 150 mM NaCl, 0.1% NP-40), PBS, and water. The peptides were eluted in 50% acetonitrile : water with 0.1% formic acid and dried in the SpeedVac.

TMT labeling

The enriched peptides were resuspended in 100 µL of 30% acetonitrile in 200 mM EPPS (pH 8.0) and labeled with 3 µL (20 µg/µL in acetonitrile) TMT reagent for 1 h at room temperature, as described above. Reactions were quenched with 3 µL of 5% Hydroxylamine for 15 min at room temperature and acidified with 5 µL of formic acid. Labeled peptides from all samples were then pooled and dried in the SpeedVac. Desalting, high-pH fractionation, and LC-MS/MS/MS analysis were performed as described above, with the IA-DTB modification (+398.25292 Da) included as a variable Cysteine modification during the searches.

Data processing and analysis for TMT-ABPP

Data quality filters

Peptides were required to be quantified in at least two biological replicates, and proteins to have at least two quantified peptides. Technical replicates were averaged before filtering. A percent-control value was discarded when the mean signal intensity of its channel was below 5,000 and the corresponding M0 (control) channel was also below 5,000. Residues quantified in exactly two replicates were excluded from analysis if at least one replicate showed less than a 1.5-fold change and the two replicates differed from each other by 2-fold or more. Proteins manually annotated as low quality were excluded.

Reactivity-change criteria

A cysteine was called a reactivity change when all of the following held. Throughout, a cysteine's "ratio to another cysteine" means its largest fold-difference from other cysteine quantified on the same protein in that condition.

1. At least one cysteine on the protein fell within 1.5-fold of the protein's expression value, or the protein carried cysteines both more than 1.5-fold above and more than 1.5-fold below it. A cysteine whose own ratio to another cysteine exceeded 3-fold satisfied this criterion regardless.

bioRxiv preprint doi: https://doi.org/10.64898/2026.09.25.754469; this version posted October 1, 2026. The copyright holder for this preprint (which was not certified by peer review) is the author/funder, who has granted bioRxiv a license to display the preprint in perpetuity. It is made available under a CC-BY 4.0 International license.

2. At least one cysteine on the protein changed less than 2-fold, or the cysteine's ratio to another cysteine exceeded 3-fold.
3. The cysteine's ratio to another cysteine exceeded 2-fold in at least two replicates. Where a peptide was missing from a replicate, its mean value across the remaining replicates was substituted.
4. Where whole-proteome expression data were available, the cysteine's ratio to the protein's expression value exceeded 2-fold.
5. For proteins with two to four peptides, the cysteine's ratio to another cysteine exceeded 2-fold. For proteins with five or more peptides – and only when at least one cysteine on the protein changed less than 2-fold – the cysteine instead differed by more than 2-fold from the protein's median cysteine value. Where expression data were available, this difference was required to be in the same direction as the cysteine's ratio to expression.

Cysteine reactivity changes on proteins with fewer than five peptides and no expression data could not be attributed to an individual residue; these were reported as ambiguous groups (Cys1;Cys2) and counted as a single reactivity change.

Proteomic platforms: Phosphoproteomics

Sample preparation

Four 6-plex experiments were performed with primary human macrophages from different donors, consisting of technical triplicates from M0 and TLR4-stimulated macrophages. Frozen cell pellets were thawed on ice and resuspended in 8 M urea lysis buffer (8 M urea in 10 mL of 200 mM EPPS pH 8.5 with 1 tablet each of Roche cOmplete Mini, EDTA-free protease inhibitor and PhosSTOP (Roche, 4906845001). Cells were then lysed with sonication (three cycles of 30 x 1 s pulses on a 80% duty cycle). Samples were then passed 10 times through a 25G needle. Protein concentration was determined with the BCA assay, and lysates were normalized to 3.7 mg/mL.

For each sample, 540 µL (2 mg) of lysate was transferred into 2 mL low-bind Eppendorf tubes and reduced with 20 µL of 300 mM DTT at 65 °C for 15 min. Proteins were then alkylated with 40 µL of 750 mM iodoacetamide for 30 min at 37 °C. Proteins were then precipitated by adding 600 µL ice-cold methanol and 120 µL chloroform to each tube, vortexing, and centrifuging (10,000 x *g*, 10 min, 4 °C). The supernatant was removed without disturbing the protein disc, and an additional 400 µL of cold methanol was then added to each sample. The pellets were resuspended by sonication, centrifuged (10,000 x *g*, 10 min, 4 °C), and resuspended by sonication in 800 µL of 200 mM EPPS (pH 8.5). Samples were digested with 5 µg of Trypsin/LysC resuspended at 0.5 µg/µL in Resuspension Buffer (Promega) in the presence of 8 µL 100 mM $CaCl_2$ at 37 °C overnight with shaking.

The next day, samples were acidified with Formic Acid until the pH was below 3. Samples were spun down (10,000 x *g*, 1 min, 25 °C) to pellet undigested precipitates, and supernatant was transferred to a new low-bind tube. C18 SepPak cartridges were conditioned with acetonitrile (3 x 1 mL) and equilibrated with Buffer A (3 x 1 mL). Samples were loaded into individual cartridges, and flow-through was loaded into the cartridge again for maximum peptide binding. Cartridges were then washed with Buffer A (3 x 1 mL) and eluted in 1 mL of Buffer B. Samples were then dried in the SpeedVac.

bioRxiv preprint doi: https://doi.org/10.64898/2026.09.25.754469; this version posted October 1, 2026. The copyright holder for this preprint (which was not certified by peer review) is the author/funder, who has granted bioRxiv a license to display the preprint in perpetuity. It is made available under a CC-BY 4.0 International license.

TiO_2 Enrichment

Samples were enriched with the High Select TiO_2 Phosphopeptide Enrichment Kit (Thermo Scientific, A32993) according to manufacturer's protocol. Briefly, all samples were resuspended in room temperature Binding/Equilibration Buffer. TiO_2 spin tips were washed with Wash Buffer (2 x 20 µL), Binding/Equilibration Buffer (2 x 20 µL), and samples were loaded twice onto the tips. Tips were then washed again with Binding/Equilibration Buffer (2 x 20 µL), Wash Buffer (2 x 20 µL), Binding/Equilibration Buffer (2 x 20 µL), Wash Buffer (2 x 20 µL), and water (1 x 20 µL). Samples were eluted twice with 50 µL Elution Buffer and dried in the SpeedVac. All flow-through and washes were saved and pooled per sample and dried in the SpeedVac.

Fe-NTA Enrichment

Flow-through and washes were enriched with the High Select Fe-NTA Phosphopeptide Enrichment Kit (Thermo Fisher, A32992) according to manufacturer's protocol. Briefly, spin columns were washed in Binding/Wash Buffer (2 x 200 µL) and the bottoms were capped with a Luer plug. Samples were resuspended in Binding/Wash Buffer and added to the column and incubated for 30 min at room temperature, with tapping every 10 minutes to resuspend resin. After incubation, samples were allowed to flow through the column, and columns were then washed with Binding/Wash Buffer (3 x 200 µL), LC-MS grade water (2 x 200 µL), and eluted twice with 100 µL of elution buffer. Samples were dried immediately in the SpeedVac.

TMT Labeling

Elutions from both Fe-NTA and TiO_2 enrichments were resuspended in 100 µL 30% ACN in 200 mM EPPS (pH 8.0), vortexed into suspension, and spun briefly (500 x *g*, 10 s, RT). 5 µL of TMT tag at 20 µg/µL was added to each channel and incubated at room temperature for 1 h. After incubation, 5 µL of 5% hydroxylamine was added to each sample to quench the reaction and incubated for 15 min at room temperature. The sample was then acidified in 8 µL of Formic Acid. Samples were then pooled and dried in the SpeedVac, with Fe-NTA and TiO_2 enrichments being kept separate. Desalting and high-pH fractionation were performed as described above.

General protocol for liquid chromatography mass spectrometry analysis of phosphoproteomics samples

TMT-labeled peptides were analyzed by LC-MS/MS/MS using a 160-min gradient on an Orbitrap Eclipse mass spectrometer (Thermo Fisher). MS1 spectra were acquired in the Orbitrap at a resolution of 120,000 over a mass range of 400-1500 m/z, with a 30% RF lens setting, automatic gain control (AGC) target set to Standard, and maximum injection time set to automatic. Monoisotopic peak determination was set to "Peptide", with the isolation window centered on the most abundant peak. Candidate precursors were required to exceed an intensity threshold of 5.0 x 10^4 and a 50% fit threshold within a 0.7 m/z fit window, and carry a charge state of 2-6. Dynamic exclusion was enabled with a repeat count of 1 and an exclusion duration of 60 s (± 10 ppm mass tolerance). The ten most abundant precursor ions from the MS1 scan were selected for MS2/MS3 analysis, isolated using the quadrupole with a 0.7 m/z isolation window and fragmented by collision-induced dissociation (CID) in the ion trap (standard AGC, 30% normalized collision energy (NCE), a 10 ms activation time, activation Q of 0.25, and a 54 ms maximum injection time). Multistage activation was enabled with a neutral loss mass of 97.9673, and resulting fragment spectra were detected in the Orbitrap at a resolution of 30,000. Synchronous precursor selection (SPS) was enabled to select up to 10 MS2 fragment ions for MS3 analysis. MS3 spectra

bioRxiv preprint doi: https://doi.org/10.64898/2026.09.25.754469; this version posted October 1, 2026. The copyright holder for this preprint (which was not certified by peer review) is the author/funder, who has granted bioRxiv a license to display the preprint in perpetuity. It is made available under a CC-BY 4.0 International license.

were acquired in the Orbitrap following high-energy collision-induced dissociation (HCD) with 65% NCE, a normalized AGC target of 200%, a maximum injection time of 200 ms, a resolution of 50,000, and an 0.7 m/z isolation window for MS3.

Data analysis and processing

Differential phosphorylation of serine, threonine, and tyrosine (S/T/Y) was searched allowing a maximum of 1, 2, 3, and 10 differential modifications per peptide. Peptide-spectrum matches were filtered using up to 2 missed cleavages, unique peptides only, average signal intensity > 5,000 for any pair of technical replicates, and a coefficient of variation < 0.5. Peptides from the different differential-modification searches were pooled and distinctly phosphorylated peptides (e.g., a singly- vs. doubly-phosphorylated version) were kept as separate entries and not averaged. Peptides sharing the same shortest representative sequence for a given S/T/Y residue were averaged (mean).

Channel ratios were first median-normalized across samples within each condition, so that the median signal intensity was equalized across the M0 or TLR4 samples. To isolate changes in phosphorylation from changes in protein abundance, replicate-level phosphosite ratios were then normalized to the whole proteome by dividing the channel ratio from each phosphosite by the corresponding protein's whole-proteome fold change. Phosphosites lacking whole-proteome values were dropped. Per-site $\log_2$ fold changes and t-test p-values were computed between conditions. Sites were called significantly phosphorylated at a fold change > 2 (after whole-proteome normalization) and $p < 0.05$ (independent two-sample t-test).

Western blot analysis

Frozen cell pellets were thawed on ice, resuspended in PBS with protease inhibitor cocktail (Roche cOmplete Mini, EDTA-free), and lysed by sonication (3 x 8 pulses, 60% duty cycle). Protein concentration was determined using the DC assay (Bio-Rad), and lysates were normalized to 2 mg/mL. Samples were combined with 4X Laemmli SDS sample buffer (Thermo Scientific, J60015.AD) and boiled at 95 °C for 5 min. Proteins were resolved on a 4-20% gel (Bio-Rad, 4561096 or 5678095) with 1X running buffer (Bio-Rad, 1610732) at 80 V for 20 min, followed by 150 V for 60 min or until the dye front reached the bottom of the gel. Proteins were transferred onto 0.45 μM PVDF membrane (Cytiva) using the Trans-Blot Turbo Transfer System in Transfer buffer (Bio-Rad, 10026938) with methanol in place of ethanol and using the manufacturer's standard transfer protocol. For total protein staining, membranes were washed with water and dried for 10 min at 37 °C. Membranes were then rehydrated with MeOH before incubation with total protein stain (LI-COR, 926-11011) for 10 min. Membranes were imaged on a ChemiDoc MP Imaging System (Bio-Rad) with the 680 nm channel. Membranes were then blocked for 1 h with 5% milk in TBST (Tris-buffered saline with 0.1% Tween-20). After incubation, membranes were washed once with TBST and incubated with primary antibody for either 2 h at room temperature or overnight at 4 °C. Membranes were then washed with TBST (4 x 5 min, with shaking), then incubated with either goat anti-mouse HRP (Thermo Fisher, G-21040), goat anti-rabbit HRP (Thermo Fisher, G-21234), donkey anti-sheep HRP (Invitrogen, A16041), or mouse anti-rabbit IgG HRP (CST, 3678) for 1 h at room temperature while shaking. Membranes were washed with TBST (4 x 5 min, with shaking), and imaged with substrate (Thermo Scientific, 32106X4 or 34095). Bands were quantified with both Fiji and ImageStudio (LI-COR) and normalized to Vinculin loading control where possible, or to the total protein stain.

bioRxiv preprint doi: https://doi.org/10.64898/2026.09.25.754469; this version posted October 1, 2026. The copyright holder for this preprint (which was not certified by peer review) is the author/funder, who has granted bioRxiv a license to display the preprint in perpetuity. It is made available under a CC-BY 4.0 International license.

Modified Western blot for S100A4

For S100A4 blots, the following changes to the standard protocol were made. The proteins were resolved on a 16.5% Criterion Tris-Tricine gel (Bio-Rad, 3450065) at 80 V for 20 min and then at 150 V until the dye front reached the bottom (2-3 h) in Tris-Tricine running buffer. The gel was then transferred to a 0.2 µM nitrocellulose membrane in a wet transfer tank for 60 min at 90 V in the cold room, using a modified transfer buffer (20% Methanol in Tris-Glycine buffer). The membrane was then blocked and stained as described above.

Marker	Species	Company	Catalog #	Clone	Dilution
Vinculin	Mouse	Santa Cruz	sc-73614	7F9	1:200
STAT4	Rabbit	Cell Signaling Technologies	2653S	C46B10	1:1000
SCO2	Rabbit	Proteintech	21223-1-AP	Polyclonal	1:200
CAMK1	Sheep	R&D Systems	AF7899	Polyclonal	1:200
S100A4	Sheep	R&D Systems	AF4138	Polyclonal	1:200
Tbck (Fy1)	Rabbit	Atlas Antibodies	HPA039951	Polyclonal	1:1000
PPP1R21 (Fy2)	Mouse	Schuhmacher et al[6] and Quentin et al[7]	--	--	1:1000
C1orf43 (Fy3)	Rabbit	Atlas Antibodies	HPA037871	Polyclonal	1:1000
Cryzl1 (Fy4)	Rabbit	Schuhmacher et al[6] and Quentin et al[7]	--	--	1:7000
DISC1	Mouse	Santa Cruz	sc-365591	B-7	1:200

Western blot - ABPP analysis

Frozen cell pellets were thawed on ice, resuspended in PBS, and lysed by sonication (3 x 8 pulses, 60% duty cycle). Protein concentration was determined using a bicinchoninic acid (microBCA) assay (Pierce, 23235), and lysates were normalized to 2 mg/mL. Methyl-PEG-Maleimide Reagent (Thermo Fisher Scientific, 22713; $MM(PEG)_{24}$) was dissolved in DMSO at 250 mM. The metabolites, GTP (Aaron Chemicals, AR00I7U9) and ATP (Aaron Chemicals, AR00I7HK), were dissolved in H_2O at 125 mM and adjusted to pH 7.5. Each metabolite and H_2O control (2 µL) were added to 46 µL of macrophage lysates, mixed by pipetting, and incubated for 10 min at room temperature. Following this pre-treatment, 2 µL of maleimide probe or DMSO was added to each sample, mixed by pipetting, and incubated for 30 min at room temperature. Samples were then combined with 4X Laemmli SDS sample buffer (Thermo Scientific, J60015.AD) and boiled at 95 °C for 10 min. Proteins were resolved on a 12% gel (Bio-Rad, 4561043) with 1X running buffer (Bio-Rad, 1610732) at 90 V for 15 min, followed by 150 V for 60 min or until the dye front reached the bottom of the gel. Proteins were transferred onto 0.45 µM PVDF membrane (Cytiva) using the Trans-Blot Turbo Transfer System in Transfer buffer (Bio-Rad, 10026938), with methanol in place of ethanol, using the manufacturer's standard transfer protocol. For total protein staining, membranes were washed with water and dried for 10 min at 37 °C. Membranes were then rehydrated with MeOH before incubation with total protein stain (LI-COR, 926-11011) for 10 min. Membranes were imaged on a ChemiDoc MP Imaging System (Bio-Rad) with the IRDye 680 setting. Membranes were then blocked for 1 h with 5% milk in TBST (Tris-buffered saline with

bioRxiv preprint doi: https://doi.org/10.64898/2026.09.25.754469; this version posted October 1, 2026. The copyright holder for this preprint (which was not certified by peer review) is the author/funder, who has granted bioRxiv a license to display the preprint in perpetuity. It is made available under a CC-BY 4.0 International license.

0.1% Tween-20). After incubation, membranes were washed once with TBST and incubated with primary antibody (Cell Signaling Technology, #2466) for either 2 h at room temperature or overnight at 4 °C. Membranes were then washed with TBST (4 x 5 min, with shaking), then incubated with goat anti-rabbit HRP (Thermo Fisher, G-21234) for 1 h at room temperature while shaking. Membranes were washed with TBST (4 x 5 min, with shaking), and imaged with substrate (Thermo Scientific, 32106X4 or 34095). Bands were quantified with both Fiji and ImageStudio Lite version 5.2 (LI-COR).

FERRY Immunoprecipitation for Western blot and mass spectrometry

Cell lysis and antibody enrichment

Frozen cell pellets were thawed on ice, resuspended in lysis buffer (50 mM Tris pH 7.4, 150 mM NaCl, 1% Triton X-100), and incubated at 4 °C with rotation. Tubes were then centrifuged to remove debris (16,000 x *g*, 4 min, 4 °C), and the supernatant was transferred to another tube. Protein concentration was determined by DC assay, and lysates were normalized to 2 mg/mL with lysis buffer. Lysate (500 µL, 1 mg) was transferred into separate low-binding Eppendorf tubes for each treatment condition. Primary antibodies (Fy2 or Fy1 (Atlas Antibodies, HPA039951); 1.5 µg/tube) or Normal IgG (CST, Rabbit (2729) or Mouse (68860)) were then added to the lysate, and the samples were incubated at 4 °C for 3 h with rotation.

Dynabead incubation and bead washes

After 3 h, 20 µL of washed Dynabeads Protein A (for rabbit antibodies, Invitrogen, 10002D) or Protein G (for mouse antibodies, Invitrogen, 10003D) were added per sample, and the samples were rotated for another 45 min. Tubes were spun briefly (500 x *g*, 5 s, 25 °C) to remove any remaining liquid from the lids and transferred to a magnetic rack. The flow-through was saved to validate target protein depletion. Beads were then washed 4 times with wash buffer (50 mM Tris pH 7.4, 150 mM NaCl, 0.2% Triton X-100). Samples prepared for Western blot analysis were then incubated with 1X Laemmli SDS sample buffer diluted in PBS for 10 min at 95 °C. Samples were spun briefly (500 x *g*, 5 s, 25 °C), placed on the magnet, and the supernatant was transferred to a new low-bind Eppendorf tube and stored at 4 °C until further analysis.

Samples prepared for downstream mass spectrometry analysis were washed an additional two times with 200 mM EPPS (pH 8.0). Samples were then eluted with 60 µL of 8 M urea in 200 mM EPPS (pH 8.0) and heated at 65 °C for 10 min. Samples were placed on the magnet, and the supernatant was transferred to a new low-bind Eppendorf tube. Freshly made DTT (3.75 µL, 200 mM in water) was added to each sample and incubated for 15 min at 65 °C. Freshly made iodoacetamide (3.75 µL, 400 mM in water) was then added and the samples were incubated at 37 °C in the shaker for 30 min. Samples were then diluted with 202.5 µL of EPPS (pH 8.0) and digested with 4 µg of Trypsin/LysC and 2 µL of 100 mM $CaCl_2$ overnight with shaking.

TMT labeling

Acetonitrile (90 µL) was added to digested samples for a final volume of 25%. TMT reagents (5 µL, 20 µg/µL) were then added to each tube and incubated for 1 h at room temperature. The reaction was quenched with 5 µL of 5% hydroxylamine for 15 min at RT, then acidified with 12 µL of formic acid. Samples were combined, split back into 2 tubes, and dried in the SpeedVac. Samples were desalted using C18 SepPak cartridges and fractionated as described above. Following fractionation, samples were combined into six final fractions and analyzed by LC-MS/MS/MS as described above.

bioRxiv preprint doi: https://doi.org/10.64898/2026.09.25.754469; this version posted October 1, 2026. The copyright holder for this preprint (which was not certified by peer review) is the author/funder, who has granted bioRxiv a license to display the preprint in perpetuity. It is made available under a CC-BY 4.0 International license.

Data processing and analysis

To correct for differences in pull-down efficiency between samples, signal intensities in each IP (TBCK or PPP1R21) were scaled by a per-donor normalization factor derived from the median signal of the bait protein, so that bait levels were equalized across conditions. Percent control values were calculated as a ratio to the per-donor median of the corresponding M0-IP control. Per-protein fold changes were computed as the $\log_2$-transformed ratios of mean intensities between the two conditions, and significance was assessed by an independent two-sample t-test. Proteins were called enriched when $FC > 1.5$ and $p < 0.05$.

Background contaminants were identified using the CRAPome (reprint-apms.org, v2.0)[8], querying all quantified proteins against the 716 human control experiments; a protein detected in more than 200 of these was flagged as a CRAPome hit. Enriched protein sets were restricted to non-CRAPome proteins prior to downstream GO-term enrichment.

Immunofluorescence

Coverslip Coating

12 mm round glass coverslips (#1.5, Corning) were sterilized in 70% Ethanol and transferred to each well of a 24-well plate. Coverslips were washed twice with molecular biology grade water and then incubated with 0.5 mL of Poly-*L*-Lysine (Sigma-Aldrich, 50-176-1647) resuspended at 0.1 mg/mL in molecular biology grade water for 20 min at room temperature. Coverslips were then washed twice in molecular biology grade water and transferred to a new 24-well plate to dry. After coverslips were fully dried, they were used immediately for cell plating.

Macrophage plating and polarization

Macrophages that were polarized for 6 days, washed once with PBS, and incubated with TrypLE for 30 min in a humidified 37 °C incubator. After 30 min, cells were gently scraped, transferred into a Falcon tube, and centrifuged (520 x *g*, 5 min, 25 °C). Cells were resuspended in PBS, counted, centrifuged again (520 x *g*, 5 min, 25 °C), and resuspended at 150,000 cells / mL in macrophage media containing mCSF. Cells (0.5 mL, 75,000 cells per well) were then transferred to a 24-well plate with pre-coated Poly-*L*-Lysine glass coverslips and left to adhere overnight at 37 °C in a humidified incubator. The next day, media was aspirated from the plates, and cells were re-supplemented with either macrophage media with mCSF or media with mCSF and LPS at 100 ng/mL and returned to the incubator for 24 h. Coverslips containing adherent cells were then washed twice with PBS-MC (PBS with 1 mM $MgCl_2$ and 0.1 mM $CaCl_2$) before incubation with freshly made 4% PFA (pH 7.0) in PBS-MC for 15 min at room temperature. Coverslips were then washed again with PBS-MC and kept at 4 °C until staining.

Immunofluorescence staining

Cells were permeabilized with 0.1% Triton X-100 in PBS-MC for 5 min at room temperature. Coverslips were then washed once with PBS-MC and blocked with 2% Normal goat serum (NGS, Jackson Immunology, 005-000-121) and 2% BSA in PBS-MC for 1 h at room temperature. Coverslips were then washed twice with PBS-MC and transferred to a humidified chamber with the adherent cells facing up. Staining solutions in blocking buffer supplemented with 0.1% Triton X-100 (60 µL) were gently layered onto the surface of each coverslip with a pipette, and the chamber was incubated overnight at 4 °C. The next day, the coverslips were transferred back into the 24-well plate and washed twice with PBS-MC. Cells were then incubated with fluorescently

bioRxiv preprint doi: https://doi.org/10.64898/2026.09.25.754469; this version posted October 1, 2026. The copyright holder for this preprint (which was not certified by peer review) is the author/funder, who has granted bioRxiv a license to display the preprint in perpetuity. It is made available under a CC-BY 4.0 International license.

labeled secondary antibodies in blocking buffer with 0.1% Triton X-100 for 1 h at room temperature in the dark. Coverslips were then washed twice with PBS-MC and incubated with DAPI (Invitrogen R37606, 2 drops per mL of blocking buffer) for 15 min at room temperature. Coverslips were then washed five times with PBS-MC and mounted onto glass slides with ProLong Diamond Antifade Mountant (Invitrogen, P36961). Sealed coverslips were then imaged on a Zeiss LSM980 confocal microscope with Airyscan 2 detector. All images reported here were acquired in conventional confocal mode with GaAsP-PMT (green/red/far-red dyes) or multialkali PMT-based detection for DAPI. A Plan-Apochromat 63x/1.40 NA oil-immersion objective was used with immersion oil of refractive index 1.518. Alexa Fluor 488 and Alexa Fluor 568 were excited at 488 nm and 561 nm and detected over spectral bandwidth of 498-552nm, and 568-640nm respectively; DAPI was excited at 405 nm and detected over 410-482nm. The confocal pinhole was set to 64.9 µm. Images were acquired at 16-bit, with bidirectional scanning, a frame size of 1908x1908 pixels and an optical zoom of 1X. Z-stacks of 15-40 planes were acquired to span the entire cell height. Channels were acquired sequentially to prevent spectral bleed-through. Laser power, detector gain, digital offset, pinhole diameter, scan speed, line averaging and optical zoom were kept identical across conditions.

Marker	Antibody Species	Catalog #	Dilution	Secondary Fluorophore
Fy1	Rabbit	HPA039951 (Atlas Antibodies)	1:200	Goat anti-Rabbit AF568
Fy2	Mouse	Schuhmacher et al[6] and Quentin et al[7]	1:1000	Goat anti-Mouse AF488
Fy4	Rabbit	Schuhmacher et al[6] and Quentin et al[7]	1:7000	Goat anti-Rabbit AF568
Rab5	Rabbit	3547 (CST)	1:200	Goat anti-Rabbit AF568

IF analysis

First, 3D image stacks were denoised by N2V neural network implemented in MotionTracking (https://motiontracking.mpi-cbg.de), which improved Signal-to-Noise Ratio (SNR) on ~30dB. Then 3D image stacks were deconvolved plane by plane by DeconSVD (MotionTracking) with theoretical microscope point-spread function (NA=1.4 and emission wavelength was taken from microscope metadata). Then background was subtracted[9] and fluorescent puncta were segmented as 3D objects in MotionTracking v.8.102.3. The segmentation threshold was set at 15% of detected vesicle intensity level.

Whole-cell masks were defined for the Fy2 channel by thresholding at an intensity of 1000. The number of puncta and average puncta volume were determined per cell. For endocytosis analysis, puncta with < 0.15 µm radius were filtered out and Rab5 puncta volume and number of Rab5 puncta per cell volume were calculated per cell, with at least 25 cells quantified per donor and condition across six donors. Individual cells were plotted on a superplot,[10] with the average of all cells reported per donor. Significance was calculated by a two-tailed paired t-test on the donor means, and average and SEM of six donors was plotted.

For colocalization, puncta with < 0.15 µm radius were filtered out, and colocalization was calculated as fraction of integral intensity associated with colocalized objects[11] with a 25% apparent object volume overlap cutoff. Given that deconvolution was performed in 2D (plane-by-

bioRxiv preprint doi: https://doi.org/10.64898/2026.09.25.754469; this version posted October 1, 2026. The copyright holder for this preprint (which was not certified by peer review) is the author/funder, who has granted bioRxiv a license to display the preprint in perpetuity. It is made available under a CC-BY 4.0 International license.

plane) and Z-dimension was not deconvolved, the "radius" was defined as $\sqrt{Area\_of\_object\_projection\_on\_plane\_XY/\pi}$.

Colocalization was determined separately for both markers (i.e., Rab5 overlap with Fy2 and Fy2 overlap with Rab5) on a per-cell basis, with at least 25 cells quantified per donor and condition across six donors. Individual cells were plotted on a superplot, with the average of all cells reported per donor. Significance was calculated by a two-tailed paired t-test on the donor means, and average and SEM of six donors was plotted. Per-cell measurements and donor-level statistics are provided in Data S4.

Proximity Ligation Assay

Day 6 macrophages were lifted as described above and plated on a 96-well glass bottom plate (Cellvis, P961.5HN) at 15,000 cells per well in macrophage media with mCSF and left to adhere overnight in a humidified incubator chamber at 37 °C. Media was then removed from the wells, and cells were polarized with either macrophage media with mCSF alone, or mCSF with LPS at 100 ng/mL. After 24 h incubation, the plate was washed twice with room temperature PBS-MC, and cells were fixed with 4% PFA in PBS-MC for 15 min at room temperature. Cells were then washed twice with PBS-MC and stored at 4 °C until staining.

Membrane labeling, permeabilization, and blocking

Cells were labeled with 5 µg/mL wheat germ agglutinin (WGA-AF488, Invitrogen W11261) in PBS-MC for 15 min at room temperature, washed twice with PBS-MC, and permeabilized with 0.1% Triton X-100 in PBS-MC for 5 min at room temperature. Cells were washed twice more and blocked with Duolink Blocking Solution (Sigma-Aldrich, DUO82007) for 1 h at 37 °C. Blocking solution was then removed, and primary antibodies diluted in Duolink Antibody Diluent (Sigma-Aldrich, DUO82008) were added and incubated overnight at 4 °C.

PLA probe incubation, ligation, and amplification

The next day, cells were washed twice for 5 min each in Wash Buffer A (0.01 M Tris, 0.15 M NaCl, 0.05% Tween-20). Duolink Probe Anti-Rabbit PLUS (DUO92002-100RXN) and Probe Anti-Mouse MINUS (DUO92004-100RXN) were diluted at a 1:5 ratio in Duolink Antibody Diluent and incubated for 20 min at room temperature before addition to cells (40 µL per well). Plates were incubated in a humidified chamber for 1 h at 37 °C. Cells were then washed twice with Wash Buffer A, and 40 µL of Ligation mix (ligase diluted 1:40 in 1X Ligation buffer; DUO92008-100RXN) was added to each well. The plate was incubated in a pre-heated humidity chamber for 30 min at 37 °C. Cells were then washed twice with Wash Buffer A, and 40 µL of Amplification mix (polymerase diluted 1:80 in 1X Amplification buffer) was added to each well, followed by an additional incubation in a pre-heated humidified chamber for 100 min at 37 °C.

Washing and mounting

Cells were washed twice with Wash Buffer B (0.2 M Tris, 0.1 M NaCl) for 10 min at room temperature, then washed once with 0.01X Wash Buffer B for 1 min. Cells were then mounted with ProLong Diamond Antifade Mountant with DAPI (Invitrogen, P36962).

PLA analysis

PLA samples in 96-well glass-bottom plates were imaged on the same LSM980 microscope used for immunofluorescence with acquisition settings held identical across all panels, conditions,

bioRxiv preprint doi: https://doi.org/10.64898/2026.09.25.754469; this version posted October 1, 2026. The copyright holder for this preprint (which was not certified by peer review) is the author/funder, who has granted bioRxiv a license to display the preprint in perpetuity. It is made available under a CC-BY 4.0 International license.

donors and sessions; acquisition parameters were otherwise as described for immunofluorescence above. Images were deconvolved in SVI Huygens Professional (Scientific Volume Imaging, V26.04) using the Classic Maximum Likelihood Estimation (CMLE) algorithm with a theoretical point-spread function computed from the microscopic parameters discussed above, voxel dimensions, and embedding medium refractive index (approx. 1.47). These parameters were used by the software to deduce signal-to-noise ratio and a quality threshold stopping criteria for deconvolution algorithm. Deconvolution iterations were run until the quality criterion was fulfilled. Identical deconvolution parameters were applied to all conditions and all donors.

Panel	Marker	Antibody Species	Catalog #	Dilution
1	Fy1	Rabbit	HPA039951 (Atlas Antibodies)	1:200
1	Fy2	Mouse	Schuhmacher et al[6] and Quentin et al[7]	1:1000
**2**	**Fy3**	**Rabbit**	**APrEST80927 (Atlas Antibodies)**	**1:200**
**2**	**Fy2**	**Mouse**	Schuhmacher et al[6] and Quentin et al[7]	**1:1000**
3	Fy4	Rabbit	Schuhmacher et al[6] and Quentin et al[7]	1:7000
3	Fy2	Mouse	Schuhmacher et al[6] and Quentin et al[7]	1:1000
**4**	**Rab5**	**Rabbit**	**3547 (CST)**	**1:200**
**4**	**Fy2**	**Mouse**	Schuhmacher et al[6] and Quentin et al[7]	**1:1000**
5	DISC1	Rabbit	40-6900 (Invitrogen)	1:200
5	Fy2	Mouse	Schuhmacher et al[6] and Quentin et al[7]	1:1000

Generation of reference protein tables

Reference list name	GO terms used	GO term names
Electron transport chain	GO:0022900	electron transport chain
GTPase activity	GO:0003924, GO:0003925, GO:0004663, GO:0005085, GO:0005092, GO:0005095, GO:0005096, GO:0007264,	GTPase activity, G protein activity, Rab geranylgeranyltransferase activity, guanyl-nucleotide exchange factor activity, GDP-dissociation inhibitor activity, GTPase inhibitor activity, GTPase activator activity, small GTPase-mediated signal transduction, Ras protein signal transduction, Rho protein signal transduction, Rac protein signal transduction, ARF protein signal transduction, Rab protein signal

bioRxiv preprint doi: https://doi.org/10.64898/2026.09.25.754469; this version posted October 1, 2026. The copyright holder for this preprint (which was not certified by peer review) is the author/funder, who has granted bioRxiv a license to display the preprint in perpetuity. It is made available under a CC-BY 4.0 International license.

	GO:0007265, GO:0007266, GO:0016601, GO:0032011, GO:0032482, GO:0032484, GO:0032486, GO:0032488, GO:0032794, GO:0034260, GO:0043087, GO:0043547, GO:0051020, GO:0051056, GO:0051057, GO:0051058, GO:0090630, GO:0097178	transduction, Ral protein signal transduction, Rap protein signal transduction, Cdc42 protein signal transduction, GTPase activating protein binding, negative regulation of GTPase activity, regulation of GTPase activity, positive regulation of GTPase activity, GTPase binding, regulation of small GTPase mediated signal transduction, positive regulation of small GTPase mediated signal transduction, negative regulation of small GTPase mediated signal transduction, activation of GTPase activity, ruffle assembly
Glycolysis		MSigDB HALLMARK_GLYCOLYSIS (not a GO-defined list)
Interferon gamma response		MSigDB HALLMARK_INTERFERON_GAMMA_RESPONSE (not a GO-defined list)
Mitochondrial	GO:0005739, GO:0031966, GO:0005759, GO:0098800, GO:0005758, GO:0044233, GO:0005740, GO:0042645, GO:0098799, GO:0005746	mitochondrion, mitochondrial membrane, mitochondrial matrix, inner mitochondrial membrane protein complex, mitochondrial intermembrane space, mitochondria-associated endoplasmic reticulum membrane contact site, mitochondrial envelope, mitochondrial nucleoid, outer mitochondrial membrane protein complex, mitochondrial respirasome
Mitochondrial translation	GO:0032543	mitochondrial translation
TNFα signaling via NF- κB		MSigDB HALLMARK_TNFA_SIGNALING_VIA_NFKB (not a GO-defined list)
Vesicle-mediated transport	GO:0060627, GO:0048193, GO:0006888, GO:0042147	regulation of vesicle-mediated transport, Golgi vesicle transport, endoplasmic reticulum to Golgi vesicle-mediated transport, retrograde transport, endosome to Golgi

bioRxiv preprint doi: https://doi.org/10.64898/2026.09.25.754469; this version posted October 1, 2026. The copyright holder for this preprint (which was not certified by peer review) is the author/funder, who has granted bioRxiv a license to display the preprint in perpetuity. It is made available under a CC-BY 4.0 International license.

(C) Key resources

REAGENT or RESOURCE	SOURCE	IDENTIFIER
**Western Blot, Immunofluorescence, and PLA Antibodies**		
Mouse monoclonal anti-Vinculin	Santa Cruz	Cat#: sc-73614<br>RRID: AB_1131294
Rabbit monoclonal anti-STAT4	Cell Signaling Technology	Cat#: 2653S<br>RRID: AB_2255156
Rabbit polyclonal anti-SCO2	Proteintech	Cat#: 21223-1-AP<br>RRID: AB_10694574
Sheep polyclonal anti-CAMK1	R&D Systems	Cat#: AF7899<br>RRID:AB_3644521
Sheep polyclonal anti-S100A4	R&D Systems	Cat#: AF4138<br>RRID: AB_1964705
CDC42 antibody	Cell Signaling Technology	Cat#: 2466<br>RRID: AB_2078082
Rabbit polyclonal anti-Tbck (Fy1)	Atlas Antibodies	Cat#: HPA039951<br>RRID: AB_10795300
Mouse monoclonal anti-PPP1R21 (Fy2)	Mouse	--
Rabbit polyclonal anti-C12orf4 (Fy3)	Atlas Antibodies	Cat#: HPA037871<br>RRID: AB_10670531
Rabbit monoclonal anti-Cryzl1 (Fy4)	Rabbit	--
Rabbit polyclonal anti-DISC1	Invitrogen	Cat#: 40-6900<br>RRID: AB_2533477
Mouse monoclonal anti-DISC1	Santa Cruz Biotech	Cat#: sc-365591<br>RRID: AB_10859378
Rab5 Antibody	Cell Signaling Technology	Cat#: 3547<br>RRID: AB_2300649
Goat anti-Mouse IgG (H+L) Cross-Adsorbed Secondary Antibody, HRP	Thermo Fisher Scientific	Cat#: G-21040<br>RRID: AB_2536527
Goat anti-Rabbit IgG (H+L) Cross-Adsorbed Secondary Antibody, HRP	Thermo Fisher Scientific	Cat#: G-21234<br>RRID: AB_2536530
Donkey anti-sheep HRP	Invitrogen	Cat#: A16041<br>RRID: AB_2534715
Goat anti-Rabbit IgG H+L (AF568) (secondary)	Invitrogen	Cat#: AB175471<br>RRID: AB_2576207
Goat anti-Mouse IgG H+L (AF488) (secondary)	Invitrogen	Cat#: AB150113<br>RRID: AB_2576208
**Flow Cytometry Reagents**		
Mouse monoclonal anti-CD14 Brilliant Violet 650	BD Biosciences	Cat#: 563419<br>RRID: AB_2744286
Rat monoclonal anti-CD11b Brilliant Violet 711	BD Biosciences	Cat#: 563168<br>RRID: AB_2716860
Mouse monoclonal anti-CD80 PE	BD Biosciences	Cat#: 560925

bioRxiv preprint doi: https://doi.org/10.64898/2026.09.25.754469; this version posted October 1, 2026. The copyright holder for this preprint (which was not certified by peer review) is the author/funder, who has granted bioRxiv a license to display the preprint in perpetuity. It is made available under a CC-BY 4.0 International license.

REAGENT or RESOURCE	SOURCE	IDENTIFIER
		RRID: AB_396606
Live/Dead Fixable NIR Viability Dye	Invitrogen	Cat#: L10119
Human TruStain FcX	BioLegend	Cat#: 422302
True-Stain Monocyte Blocker	BioLegend	Cat#: 426102
Paraformaldehyde (4%)	Fujifilm	Cat#: 163-20145
EDTA (0.5 M, pH 8.0)	Thermo Fisher Scientific	Cat#: AM9260G
**TLR and STING agonists**		
CU-T12-09	Millipore Sigma	Cat#: 5325830001
Poly(I:C)	Tocris Bioscience	Cat#: 42-871-0
Lipopolysaccharides from Escherichia coli O111:B4	Millipore Sigma	Cat#: L2630-10
Imiquimod	Fisher Scientific	Cat#: AAJ63990MC
CL075	Millipore Sigma	Cat#: SML2235
CpG ODN 2006	Integrated DNA technologies	--
2’3’-cGAMP	InvivoGen	Cat#: tlrl-nacga23-5
**Biological Samples**		
Human PB Leukopak, Fresh	STEMCELL Technologies	Cat#: 70500.1
Human LRS Cone	STEMCELL Technologies	Cat#: 200-0093
Human buffy coat	New York Blood Center	--
Blood and leukapheresis samples	Rockefeller University Hospital	--
**Chemicals, peptides, and recombinant proteins**		
RPMI 1640 Medium, no glutamine	Thermo Fisher Scientific	Cat#: 21870-076
Penicillin-Streptomycin (10,000 U/mL)	Thermo Fisher Scientific	Cat#: 15140-122
Sodium Pyruvate (100 mM)	Thermo Fisher Scientific	Cat#: 11360070
GlutaMAX	Thermo Fisher Scientific	Cat#: 35050061
Fetal Bovine Serum, heat inactivated	Omega Scientific	Cat#: FB-02
HEPES (pH 7.0)	Boston Bioproducts	Cat#: BBH-70
Lymphoprep	STEMCELL Technologies	Cat#: 07811
RBC Lysis Buffer, 10X	Santa Cruz Biotechnologies	Cat#: sc-296258
Human Recombinant M-CSF	STEMCELL Technologies	Cat#: 78057.2
TrypLE	Gibco	Cat#: 12604021
TRIzol Reagent	Invitrogen	Cat#: 15-596-026
cOmplete Mini Protease Inhibitor (EDTA-free)	Roche	Cat#: 11836170001

bioRxiv preprint doi: https://doi.org/10.64898/2026.09.25.754469; this version posted October 1, 2026. The copyright holder for this preprint (which was not certified by peer review) is the author/funder, who has granted bioRxiv a license to display the preprint in perpetuity. It is made available under a CC-BY 4.0 International license.

REAGENT or RESOURCE	SOURCE	IDENTIFIER
PhosSTOP	Roche	Cat#: 4906845001
Laemmli SDS sample buffer, reducing (4X)	Thermo Fisher Scientific	Cat#: J60015.AD
Precision Plus Protein Dual Color Standards	Bio-Rad	Cat#: 1610374
4–20% Criterion TGX Stain-Free Protein Gels, 26 well, 15 µL	Bio-Rad	Cat#: 5678095
4–20% Mini-PROTEAN TGX Precast Protein Gels, 15-well, 15 µL	Bio-Rad	Cat#: 4561096
12% Mini-PROTEAN® TGX™ Precast Protein Gels	Bio-Rad	Cat#: 4561043
10x Tris/Glycine/SDS	Bio-Rad	Cat#: 1610732
16.5% Criterion Tris-Tricine Gel	Bio-Rad	Cat#: 3450065
Amersham Protran 0.2 NC nitrocellulose	Cytiva	Cat#: 45-004-001
Amersham Hybond P 0.45 µM PVDF	Cytiva	Cat#: GE10600029
Extra Thick Blot Filter Paper 8.6 x 13.5 cm	Bio-Rad	Cat#: 1703967
5x TBT Transfer Buffer, 1L	Bio-Rad	Cat#: 10026938
Dry milk powder	Research Products International	Cat#: M17200-500.0
OmniPur BSA, Fraction V, Heat Shock Isolation	Sigma-Aldrich	Cat#: 2960-500GM
Revert 700 Total Protein Stain	LI-COR	Cat#: 926-11011
Pierce ECL Western Blotting Substrate	Thermo Fisher Scientific	Cat#: 32106
Urea	Supelco	Cat#: 108487
Iodoacetamide	Sigma-Aldrich	Cat#: I1149
Dithiothreitol (DTT)	Fisher Scientific	Cat#: BP172-25
Trypsin, sequencing grade	Promega	Cat#: V5111
Trypsin/Lys-C Mix, Mass Spec Grade	Promega	Cat#: V5073
Pierce™ Streptavidin Agarose	Thermo Fisher Scientific	Cat#: 20353
Acetonitrile, LC/MS grade	Fisher Scientific	Cat#: A955
Hydroxylamine solution	Sigma-Aldrich	Cat#: 467804
EPPS	Alfa Aesar	Cat#: A13714-22
TMT6plex Isobaric Label Reagent Set	Thermo Fisher	Cat#: 90068
TMT10plex Isobaric Label Reagent Set	Thermo Fisher Scientific	Cat#: 90406
TMTpro 16plex Label Reagent Set	Thermo Fisher Scientific	Cat#: A44521
Formic Acid	VWR	Cat#: 94318
NP-40	Millipore Sigma	Cat#: 492016
Methyl-PEG-Maleimide Reagent (MM(PEG)24)	Thermo Fisher Scientific	Cat#: 22713
5’-GTP trisodium salt	Aaron Chemicals	Cat#: AR00I7U9
Adenosine 5'-(tetrahydrogen triphosphate), disodium salt, hydrate	Aaron Chemicals	Cat#: AR00I7HK
Normal IgG (Rabbit)	Cell Signaling Technology	Cat#: 2729<br>RRID: AB_1031062
Normal IgG (Mouse)	Cell Signaling Technology	Cat#: 68860
Dynabeads Protein A	Invitrogen	Cat#: 10002D

bioRxiv preprint doi: https://doi.org/10.64898/2026.09.25.754469; this version posted October 1, 2026. The copyright holder for this preprint (which was not certified by peer review) is the author/funder, who has granted bioRxiv a license to display the preprint in perpetuity. It is made available under a CC-BY 4.0 International license.

REAGENT or RESOURCE	SOURCE	IDENTIFIER
Dynabeads Protein G	Invitrogen	Cat#: 10003D
Glass #1.5 Round Coverslips, 12mm	Fisher Scientific	Cat#: 1254581
Superfrost Microscope Slides	Fisher Scientific	Cat#: 22042944
Paraformaldehyde	Sigma-Aldrich	Cat#: P6148
Normal Goat Serum (NGS)	Jackson Immunology	Cat#: 005-000-121
NucBlue™ Fixed Cell ReadyProbes™ Reagent (DAPI)	Invitrogen	Cat#: R37606
ProLong Diamond Antifade Mountant	Invitrogen	Cat#: P36961
96-Well Glass Bottom Plate	Cellvis	Cat#: P961.5HN
Wheat Germ Agglutinin (WGA-AF488)	Invitrogen	Cat#: W11261
ProLong Diamond Antifade Mountant with DAPI	Invitrogen	Cat#: P36962
Poly-*L*-Lysine Hydrobromide	Sigma-Aldrich	Cat#: P6282
**Critical commercial assays**		
DC Protein Assay Kit	Bio-Rad	Cat#: 5000111
EasySep Monocyte Enrichment Kit (without CD16 Depletion)	STEMCELL Technologies	Cat#: 19058
IL-6 DuoSet ELISA Kit	R&D Systems	Cat#: DY206
TNFα DuoSet ELISA Kit	R&D Systems	Cat#: DY210
Direct-zol RNA Miniprep Kit	Zymo Research	Cat#: R2052
Qubit RNA Broad Range (BR) Kit	Invitrogen	Cat#: Q10210
iScript cDNA Synthesis Kit	Bio-Rad	Cat#: 1708891
SYBR Select Master Mix	Applied Biosystems	Cat#: 4472908
High Select TiO_2 Phosphopeptide Enrichment Kit	Thermo Scientific	Cat#: A32993
High Select Fe-NTA Phosphopeptide Enrichment Kit	Thermo Fisher	Cat#: A32992
Pierce C18 pipette tips	Thermo Fisher Scientific	Cat#: 87784
Sep-Pak Vac C18 cartridges	Waters	Cat#: WAT054955
Micro BCA Protein Assay Kit	Thermo Fisher Scientific	Cat#: 23235
Brilliant Stain Buffer	BD Biosciences	Cat#: 566349
UltraComp eBeads Plus Compensation Beads	Invitrogen	Cat#: 01-3333-42
Duolink Blocking Solution	Sigma-Aldrich	Cat#: DUO82007
Duolink Antibody Diluent	Sigma-Aldrich	Cat#: DUO82008
Duolink Probe Anti-Rabbit PLUS	Sigma-Aldrich	Cat#: DUO92002-100RXN
Duolink Probe Anti-Mouse MINUS	Sigma-Aldrich	Cat#: DUO92004-100RXN
Duolink In Situ Detection Reagents Red	Sigma-Aldrich	Cat#: DUO92008-100RXN
Illumina stranded mRNA kit	Illumina	Cat#: 20040534
**Software and algorithms**		

bioRxiv preprint doi: https://doi.org/10.64898/2026.09.25.754469; this version posted October 1, 2026. The copyright holder for this preprint (which was not certified by peer review) is the author/funder, who has granted bioRxiv a license to display the preprint in perpetuity. It is made available under a CC-BY 4.0 International license.

REAGENT or RESOURCE	SOURCE	IDENTIFIER
RAW Converter	version 1.1.0.22; 2004 release	fields.scripps.edu/rawconv
Integrated Proteomics Pipeline (IP2) and ProLuCID	Integrated Proteomics Applications, Inc	goldfish.scripps.edu
Prism (v10)	GraphPad Software Inc	graphpad.com/scientific-software/prism
FlowJo (v10.8.2)	Treestar Inc.	flowjo.com
ImageJ	NIH	imagej.nih.gov/ij
MotionTracking	Version 8.102.3	zeriallab.org/resources/
ChimeraX	Version 1.10.1	cgl.ucsf.edu/chimerax/
Adobe Illustrator 2026	Adobe	adobe.com
STAR (v2.7.7a)	Dobin et al.[12]	github.com/alexdobin/STAR
samtools (v1.9)	Danecek et al.[13]	htslib.org
featureCounts (part of the subread package; v1.4.6-p5)	Liao et al.[14]	subread.sourceforge.net
DESeq2 (v1.30.1)	Love et al.[15]	bioconductor.org/packages/release/bioc/html/DESeq2.html
ggplot2 (v3.3.5)	Wickham, H.[16]	ggplot2.tidyverse.org
ggrepel (v0.9.1)	Slowikowski, K.[17]	github.com/slowkow/ggrepel
Python (v3.13.2)	Python Software Foundation	python.org/doc/versions
Seaborn (v0.13.2)	Waskom et al., 2021[18]	seaborn.pydata.org
Plotly (v6.1.0)	Plotly Technologies Inc. Collaborative data science. Montréal, QC, 2015. plot.ly	plotly.com/python
Matplotlib (v3.10.1)	Hunter et al., 2007[19]	matplotlib.org
Scikit-learn (v1.7.0)	Pedregosa et al., 2011[20]	scikit-learn.org/stable
Pandas (v2.2.3)	McKinney, 2010[21]	pandas.pydata.org
NumPy (v2.2.3)	Van der Walt et al., 2011[22]	numpy.org
imputeLCMD (v2.1)	Lazar et al. 2022[23]	cran.r-project.org/package=imputeLCMD
GSEApy (v1.1.9)	Fang et al. 2023[24]	github.com/zqfang/GSEApy
GOATOOLS	Klopfenstein et al. 2018[25]	github.com/tanghaibao/goatools
MassHunter Profinder software v.10.0	Agilent Technologies, Inc.	www.agilent.com/en/product/software-informatics/mass-spectrometry-

bioRxiv preprint doi: https://doi.org/10.64898/2026.09.25.754469; this version posted October 1, 2026. The copyright holder for this preprint (which was not certified by peer review) is the author/funder, who has granted bioRxiv a license to display the preprint in perpetuity. It is made available under a CC-BY 4.0 International license.

REAGENT or RESOURCE	SOURCE	IDENTIFIER
		software/data-analysis/mass-profiler-professional-software
MS-DIAL software [26]	Tsugawa et al. 2015[26]	systemsomicslab.github.io/compms/msdial/main.html
AlphaFold3 (AF3, version 3.0.0)	Abramson et al. 2024[27]	github.com/google-deepmind/alphafold3

D) References

1. Gu, Z. Complex heatmap visualization. *Imeta* **1**, e43 (2022).

2. Vinogradova, E.V. *et al.* An Activity-Guided Map of Electrophile-Cysteine Interactions in Primary Human T Cells. *Cell* **182**, 1009–1026.e1029 (2020).

3. Kojima, H. *et al.* A post-translational regulatory map of chronic antigen-driven human T cell dysfunction. *bioRxiv* (2026).

4. Buitinck, L. *et al.* API design for machine learning software: experiences from the scikit-learn project. *arXiv*; 2013.

5. Mathieson, T. *et al.* Systematic analysis of protein turnover in primary cells. *Nature Communications* **9**, 689 (2018).

6. Schuhmacher, J.S. *et al.* The Rab5 effector FERRY links early endosomes with mRNA localization. *Molecular Cell* **83**, 1839–1855.e1813 (2023).

7. Quentin, D. *et al.* Structural basis of mRNA binding by the human FERRY Rab5 effector complex. *Molecular Cell* **83**, 1856–1871.e1859 (2023).

8. Mellacheruvu, D. *et al.* The CRAPome: a contaminant repository for affinity purification-mass spectrometry data. *Nature Methods* **10**, 730–736 (2013).

9. Kalaidzidis, Y., Morales-Navarrete, H., Kalaidzidis, I. & Zerial, M. Intracellular Background Estimation for Quantitative Fluorescence Microscopy. *Proceedings* **33** (2019).

10. Lord, S.J., Velle, K.B., Mullins, R.D. & Fritz-Laylin, L.K. SuperPlots: Communicating reproducibility and variability in cell biology. *Journal of Cell Biology* **219** (2020).

11. Kalaidzidis, Y., Kalaidzidis, I. & Zerial, M. A probabilistic method to quantify the colocalization of markers on intracellular vesicular structures visualized by light microscopy. *AIP Conference Proceedings* **1641** (2015).

bioRxiv preprint doi: https://doi.org/10.64898/2026.09.25.754469; this version posted October 1, 2026. The copyright holder for this preprint (which was not certified by peer review) is the author/funder, who has granted bioRxiv a license to display the preprint in perpetuity. It is made available under a CC-BY 4.0 International license.

12. Dobin, A. *et al.* STAR: ultrafast universal RNA-seq aligner. *Bioinformatics* **29**, 15–21 (2013).

13. Danecek, P. *et al.* Twelve years of SAMtools and BCFtools. *Gigascience* **10** (2021).

14. Liao, Y., Smyth, G.K. & Shi, W. featureCounts: an efficient general purpose program for assigning sequence reads to genomic features. *Bioinformatics* **30**, 923–930 (2014).

15. Love, M.I., Huber, W. & Anders, S. Moderated estimation of fold change and dispersion for RNA-seq data with DESeq2. *Genome Biology* **15**, 550 (2014).

16. Wickham, H. *ggplot2: Elegant Graphics for Data Analysis*. Springer: Cham, 2016.

17. Slowikowski, K. ggrepel: Automatically Position Non-Overlapping Text Labels with 'ggplot2'. 0.9.1 ed: Comprehensive R Archive Network (CRAN); 2021.

18. Waskom, M.L. seaborn: statistical data visualization. *The Journal of Open Source Software* **6**, 3021 (2021).

19. Hunter, J.D. Matplotlib: A 2D Graphics Environment. *Computing in Science & Engineering* **9**, 90–95 (2007).

20. Pedregosa, F. *et al.* Scikit-learn: Machine Learning in Python. *Journal of Machine Learning Research* **12**, 2825–2830 (2011).

21. McKinney, W. Data Structures for Statistical Computing in Python. *Proceedings of the 9th Python in Science Conference*, 56–61 (2010).

22. Walt, S.v.D., Colbert, S.C. & Varoquaux, G. The NumPy array: a structure for efficient numerical computation. *Computing in Science and Engineering* **13**, 22–30 (2011).

23. Lazar, C., Burger, T. & Wieczorek, S. imputeLCMD: A Collection of Methods for Left Censored Missing Data Imputation. 2.1 ed: Comprehensive R Archive Network (CRAN); 2022.

24. Fang, Z., Liu, X. & Peltz, G. GSEApy: a comprehensive package for performing gene set enrichment analysis in Python. *Bioinformatics* **39** (2023).

25. Klopfenstein, D.V. *et al.* GOATOOLS: A Python library for Gene Ontology analyses. *Sci Rep* **8**, 10872 (2018).

26. Tsugawa, H. *et al.* MS-DIAL: data-independent MS/MS deconvolution for comprehensive metabolome analysis. *Nature Methods* **12**, 523–526 (2015).

bioRxiv preprint doi: https://doi.org/10.64898/2026.09.25.754469; this version posted October 1, 2026. The copyright holder for this preprint (which was not certified by peer review) is the author/funder, who has granted bioRxiv a license to display the preprint in perpetuity. It is made available under a CC-BY 4.0 International license.

27. Abramson, J. *et al.* Accurate structure prediction of biomolecular interactions with AlphaFold 3. *Nature* **630**, 493–500 (2024).
